# Association of Genetic Variants with Isolated Fasting Hyperglycaemia and Isolated Postprandial Hyperglycaemia in a Han Chinese Population

**DOI:** 10.1371/journal.pone.0071399

**Published:** 2013-08-19

**Authors:** Xiaomu Kong, Jing Hong, Ying Chen, Li Chen, Zhigang Zhao, Qiang Li, Jiapu Ge, Gang Chen, Xiaohui Guo, Juming Lu, Jianping Weng, Weiping Jia, Linong Ji, Jianzhong Xiao, Zhongyan Shan, Jie Liu, Haoming Tian, Qiuhe Ji, Dalong Zhu, Zhiguang Zhou, Guangliang Shan, Wenying Yang

**Affiliations:** 1 Department of Endocrinology, Key Laboratory of Diabetes Prevention and Control, China-Japan Friendship Hospital, Beijing, China; 2 Department of Bioinformatics, Beijing Genetics Institute, Shenzhen, Guangdong, China; 3 Department of Endocrinology, Qilu Hospital of Shandong University, Jinan, Shandong, China; 4 Department of Endocrinology, Henan Provincial People's Hospital, Zhengzhou, Henan, China; 5 Department of Endocrinology, Second Affiliated Hospital of Harbin Medical University, Harbin, Heilongjiang, China; 6 Department of Endocrinology, Xinjiang Uygur Autonomous Region's Hospital, Urmqi, Xinjiang, China; 7 Department of Endocrinology, Fujian Provincial Hospital, Fuzhou, Fujian, China; 8 Department of Endocrinology, Peking University First Hospital, Beijing, China; 9 Department of Endocrinology, Chinese People's Liberation Army General Hospital, Beijing, China; 10 Department of Endocrinology, Third Affiliated Hospital of Sun Yat-sen University, Guangzhou, Guangdong, China; 11 Department of Endocrinology, Shanghai Jiaotong University Affiliated Sixth People's Hospital, Shanghai, China; 12 Department of Endocrinology, Peking University People's Hospital, Beijing, China; 13 Department of Endocrinology, First Hospital of China Medical University, Shenyang, Liaoning, China; 14 Department of Endocrinology, Shanxi Provincial People's Hospital, Taiyuan, Shanxi, China; 15 Department of Endocrinology, West China Hospital of Sichuan University, Chengdu, Sichuan, China; 16 Department of Endocrinology, Xijing Hospital of Fourth Military Medical University, Xi'an, Shaanxi, China; 17 Department of Endocrinology, Affiliated Drum Tower Hospital of Nanjing University Medical School, Nanjing, Jiangsu, China; 18 Department of Endocrinology, Xiangya Second Hospital, Changsha, Hunan, China; 19 Department of Epidemiology, Peking Union Medical College, Beijing, China; Tulane School of Public Health and Tropical Medicine, United States of America

## Abstract

**Background:**

Though multiple single nucleotide polymorphisms (SNPs) associated with type 2 diabetes have been identified, the genetic bases of isolated fasting hyperglycaemia (IFH) and isolated postprandial hyperglycaemia (IPH) were still unclear. In present study, we aimed to investigate the association of genome-wide association study-validated genetic variants and IFH or IPH in Han Chinese.

**Methods/Principal Findings:**

We genotyped 27 validated SNPs in 6,663 unrelated individuals comprising 341 IFH, 865 IPH, 1,203 combined fasting hyperglycaemia and postprandial hyperglycaemia, and 4,254 normal glycaemic subjects of Han ancestry. The distributions of genotype frequencies of *FTO*, *CDKAL1* and *GCKR* were significant different between individuals with IFH and those with IPH (SNP(*p_trend_*): rs8050136(0.0024), rs9939609(0.0049), rs7756992(0.0122), rs780094(0.0037)). Risk allele of *FTO* specifically increased the risk of IFH (rs8050136: OR 1.403 [95% CI 1.125–1.750], *p* = 0.0027; rs9939609: 1.398 [1.120–1.744], *p* = 0.0030). G allele of *CDKAL1* specifically increased the risk of IPH (1.217 [1.092–1.355], *p* = 0.0004). G allele of *GCKR* increased the risk of IFH (1.167 [0.999–1.362], *p* = 0.0513), but decreased the risk of IPH (0.891 [0.801–0.991], *p* = 0.0331). In addition, *TCF7L2* and *KCNQ1* increased the risk of both IFH and IPH. When combined, each additional risk allele associated with IFH increased the risk for IFH by 1.246-fold (*p*<0.0001), while each additional risk allele associated with IPH increased the risk for IPH by 1.190-fold (*p*<0.0001).

**Conclusion/Significance:**

Our results indicate that genotype distributions of variants from *FTO, GCKR*, *CDKAL1* were different between IPH and IFH in Han Chinese. Variants of genes modulating insulin sensitivity (*FTO*, *GCKR*) contributed to the risk of IFH, while variants of genes related to beta cell function (*CDKAL1*) increase the risk of IPH.

## Introduction

The number of people with diabetes grows worldwide. In the past few decades, China has experienced a dramatic increase in diabetes incidence. According to the recent Chinese National Diabetes and Metabolic Disorders Study (CNDMDS) performed in 2007–2008, about 92.4 million adults (9.7% of the adult population) in China have diabetes [1]. Type 2 diabetes is the most common type of diabetes, caused by an interaction of multiple genetic and environmental factors.

Newly diagnosed diabetes generally includes IFH, IPH or combined fasting and postprandial hyperglycaemia (FH/PH) [2]. According to the DECODE study [3], IFH and IPH accounted for 40.4% and 28.4% of newly diagnosed diabetes in Caucasians, respectively. In a study by Yang *et al.* [1], the prevalence of IPH was reported to be 47%, and that of IFH was 17% in newly diagnosed cases of diabetes, indicating IPH was the major type of diabetes in Han Chinese, which is a markedly different profile compared to Caucasians.

It was reported that IFH and IPH are derived from isolated impaired fasting glucose and isolated impaired glucose tolerance respectively [4]. Though the etiology of IFH and IPH is unclear, their heterogeneity in clinical manifestation has been well described. A higher level of insulin resistance within the liver is associated with IFH; whereas, impairment of early phase insulin secretion and total insulin secretion in response to glucose were worse in subjects with IPH [2]. Also, there were significant differences in duration, drug therapy, chronic complications and mortality between IFH and IPH subjects [5]. According to the DECODE study [3], postprandial glucose concentrations of subjects with type 2 diabetes were found to be positively associated with incidence of cardiovascular disease, independent of fasting glucose. However, fasting hyperglycaemia was not found to be predictive of the incidence of cardiovascular disease in patients with type 2 diabetes. Therefore, we speculate that IFH and IPH have different genetic etiology.

It has been reported that all subjects with newly diagnosed IFH or IPH ultimately develop fasting hyperglycaemia combined with postprandial hyperglycaemia over the course of decades [4]. In addition, type 2 diabetes patients recruited in hospital-based studies cannot be distinguished to have IFH or IPH when they are first diagnosed. Over the past decade, many studies have attempted to elucidate susceptibility genes for type 2 diabetes. To date, more than 50 genes have been found to be involved in the pathogenesis of type 2 diabetes, mostly in Caucasian populations. However, no susceptible genetic loci of IFH or IPH have been investigated due to difficulties in sampling. It is worthwhile to determine whether common genetic variations play a role in their pathogenesis, as well as to distinguish the common and different genetic basis of IFH and IPH.

In this study, we examined the association of 27 SNPs in GWAS-validated type 2 diabetes susceptible variants [6]–[18] in patients with IPH or IFH. The study population was a nationally representative cohort of newly diagnosed patients of Han ancestry recruited during CNDMDS conducted in 2007–2008. Further, we compared the difference of genetic basis of IFH and IPH.

## Methods

### Ethics Statement

The study protocol was approved by the Ethics Committee of China-Japan Friendship Hospital in Beijing and was in accordance with Helsinki Declaration II. Written informed consent was obtained from all participants before data collection.

### Participants

All samples were recruited from CNDMDS [1]. After the exclusion of subjects with type 1 diabetes and special type diabetes, a total of 6,663 unrelated Han people from 13 provinces and municipalities participated in the final analysis, including 341 with IFH, 865 with IPH, 1,203 with combined FH/PH, and 4,254 control subjects. Each participant received a standard 75 g oral glucose tolerance test (OGTT). Type 2 diabetes was defined by 1999 WHO criteria. IFH was defined as fasting plasma glucose (FPG) ≥7 mmol/l (126 mg/dl) and 2-hour plasma glucose in OGTT (2 h PG) <11.1 mmol/l (200 mg/dl). IPH was defined as FPG <7.0 mmol/l and 2 h PG ≥11.1 mmol/l. Combined FH/PH was defined as FPG ≥7 mmol/l and 2 h PG ≥11.1 mmol/l. The inclusion criteria for normoglycaemic control were: 1) age over 40 years; 2) normal glucose regulation in OGTT according to WHO criteria (FPG <6.1 mmol/l (110 mg/dl) and 2 h PG <7.8 mmol/l (140 mg/dl)); 3) no diabetes history or family history of diabetes; 4) BMI <28 kg/m^2^; 5) normal blood pressure (systolic blood pressure <140 mmHg and diastolic blood pressure <90 mmHg); 6) normal blood lipid levels (triglycerides (TG) <1.7 mmol/l and high density lipoprotein-cholesterol ≥1.0 mmol/l). Clinical characteristics of the study groups are shown in Table 1. Normally distributed data are shown as mean ± SD while non-Gaussian data are shown as median and interquartile range. Non-fatal cardiovascular diseases were determined as previously reported [1].

**Table 1:** Clinical characteristics of study population.

		Isolated fasting	Isolated postprandial	All newly diagnosed
	control	hyperglycaemia	hyperglycaemia	type 2 diabetes
**Samples (** ***n*** **)**	4,254	341	865	2,409
**Male/Female (** ***n*** **)**	1,382/2,872	175/166	382/483	1,062/1,347
**Age (years)**	50.69±8.38	51.19±12.58	56.17±11.52	55.20±11.60
**BMI (kg/m^2^)**	23.07 (21.28,24.77)	25.26 (23.03,28.56)	25.77 (23.52,28.13)	25.85 (23.62,28.33)
**Fasting plasma glucose (mmol/l)**	5.02 (4.68,5.40)	7.53 (7.20,8.26)	6.00 (5.45,6.50)	7.49 (6.41,9.16)
**30min plasma glucose (mmol/l)**	8.10 (7.00,9.20)	11.10 (9.20,12.98)	11.30 (9.64,12.79)	12.27 (10.32,14.55)
**2h plasma glucose (mmol/l)**	5.76 (4.90,6.60)	8.41 (6.71,9.70)	12.61 (11.76,14.18)	13.70 (11.72,17.19)
**Fasting serum insulin (mU/l)**	6.31 (4.90,8.45)	9.10 (6.14,12.30)	8.16 (5.93,11.66)	8.80 (6.12,12.62)
**30min serum insulin (mU/l)**	33.09 (20.92,52.64)	28.18 (17.05,47.99)	25.84 (15.11,43.80)	20.67 (11.87,36.86)
**2h serum insulin (mU/l)**	22.37 (13.94,34.96)	29.26 (17.97,49.56)	50.96 (26.56,90.70)	33.44 (19.07,61.04)
**HOMA-IR**	1.40 (1.06,1.90)	3.22 (2.12,4.47)	2.15 (1.49,3.10)	3.07 (1.98,4.61)
**HOMA-B (%)**	85.55 (61.09,125.08)	42.47 (29.30, 61.20)	71.58 (47.89, 102.95)	45.27 (27.62,73.88)
**ISIm**	8.38 (6.19, 11.34)	4.35 (3.08, 6.32)	4.66 (2.97, 6.82)	4.13 (2.78, 6.04)
**Insulinogenic index (**Δ**I_30_/**Δ**G_30_)**	9.40 (5.03,17.31)	4.74 (2.10,11.68)	3.36 (1.78,6.78)	2.68 (1.29,5.98)
**Prevalence of cardiovascular diseases (%, ** ***n_total_*** **/** ***n_affected_*** **)**	3.25%, 3,422/115	6.72%, 250/18	11.72%, 625/83	11.18%, 1,967/220

Data are shown as mean ± SD for normally distributed values, median (interquartile range) for non-normally distributed values, or *n* (%).

### Clinical examinations

Anthropometric and biochemical characteristics were carefully examined. Height, weight, waist circumference (WC) and hip circumference were measured with subjects lightly clothed, from which BMI and waist-hip-ratio were calculated. Without glucose-lowering treatment, subjects were requested to fast from food for more than 10 hours and given a standard 75 g OGTT the next day. Blood samples were drawn at 0, 30 and 120 minutes after OGTT to measure plasma glucose and serum insulin concentrations. Serum insulin was measured by double-antibody radioimmunoassay. HOMA was used to estimate insulin resistance (HOMA-IR: fasting serum insulin (mU/l) × FPG (mmol/l)/22.5) and beta cell function (HOMA-B: fasting serum insulin (mU/l) ×20/(FPG (mmol/l) –3.5)). Insulinogenic index (ΔI_30_/ΔG_30_) was calculated from fasting and 30-min serum insulin (mU/l) and plasma glucose (mmol/l) during OGTT using (Ins_30_ – Ins_0_)/(Glu_30_ – Glu_0_). The compensation of beta cells to insulin resistance was calculated using ΔI_30_/ΔG_30_/HOMA-IR. Matsuda index (ISIm) was calculated as 10,000/(Glu_0_ (mg/dl) × Ins_0_ (mU/l) × mean glucose OGTT (mg/dl) × mean insulin (mU/l))^1/2^.

Total cholesterol, TG, high density lipoprotein-cholesterol and low density lipoprotein-cholesterol in fasting serum were tested using an automatic biochemical analyzer (Olympus, Tokyo, Japan).

**Figure 2:**
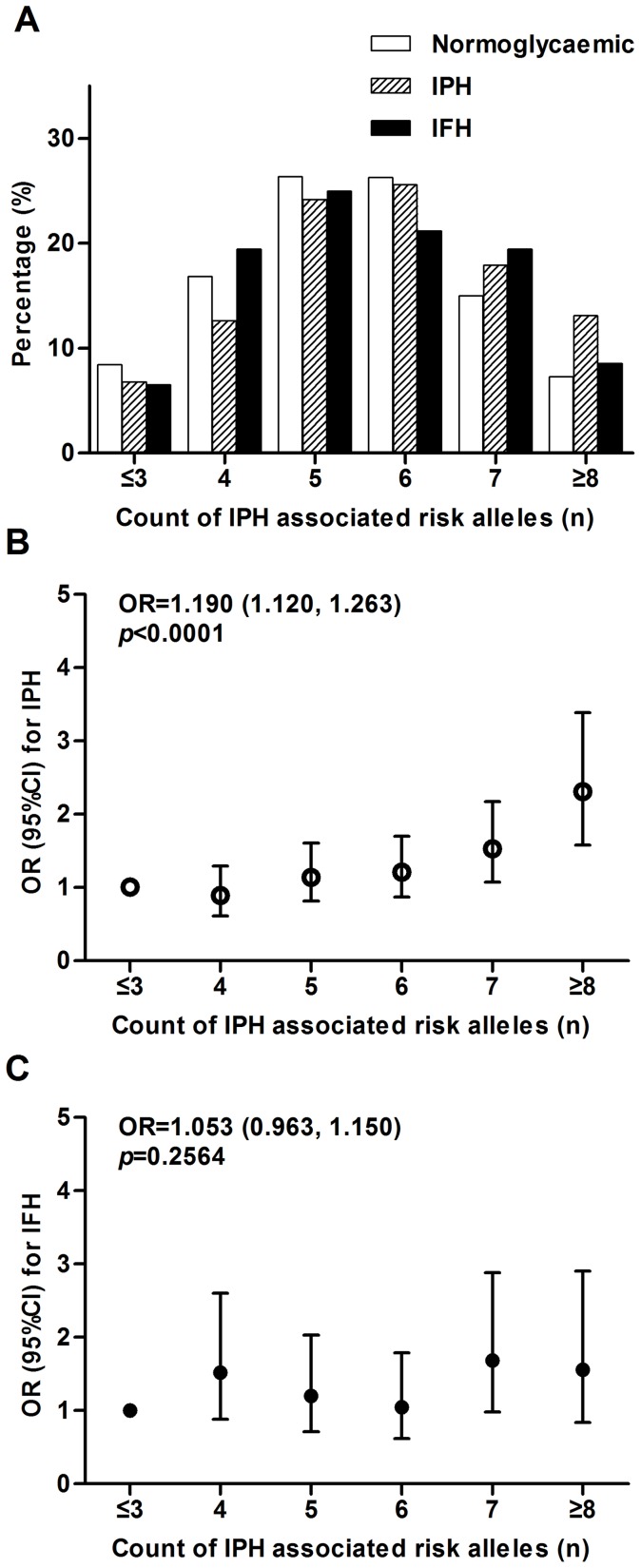
Combined effects of increasing numbers of isolated postprandial hyperglycaemia-associated risk alleles from *TCF7L2*, *CDKAL1*, *KCNQ1*, *PRC1*, *TP53INP1* and *GCKR*. A: The isolated postprandial hyperglycaemia-associated risk allele distribution in controls, participants with isolated fasting hyperglycaemia and isolated postprandial hyperglycaemia. Each additional risk allele increased the risk of isolated postprandial hyperglycaemia by 1.19-fold (*p*<0.0001) (B) but not the risk of isolated fasting hyperglycaemia (C). OR and 95% CI were plotted on the y-axis for the corresponding number of risk alleles on the x-axis (black circles  =  risk of isolated fasting hyperglycaemia; white circles  =  risk of isolated postprandial hyperglycaemia). B: Participants harboring eight or more risk alleles had a 2.31-fold increased risk for isolated postprandial hyperglycaemia (*p*<0.0001) compared with the reference group. C: However, risk for isolated fasting hyperglycaemia was not increased as risk allele number increasing. IFH, isolated fasting hyperglycemia; IPH, isolated postprandial hyperglycemia.

Joint study of IPH associated SNPs, including *TCF7L2*, *CDKAL1*, *KCNQ1*, *PRC1, TP53INP1* and *GCKR*, shows that individuals with IPH carry more risk alleles from the above loci than controls (*p_trend_* <0.0001; Figure 2A). Risk of IPH increased 19.0% for each additional risk allele carried (1.190 [1.120–1.263] per allele, *p*<0.0001; Figure 2B). In SNPs associated with IPH, the numbers of risk alleles carried in IFH subjects were not significantly more than that in the control group (*p_trend_*  = 0.2752; Figure 2A); and the number of risk alleles did not increase the risk of IFH (1.053 [0.963–1.150] per allele, *p* = 0.2564; Figure 2C).

### Genotyping

Genomic DNA samples were isolated from the peripheral blood using a DNA extraction kit. Genotyping was accomplished with Illumina GoldenGate Indexing assay (Illumina Inc., San Diego, USA), used according to the manufacturer's instructions. We selected 31 common SNPs from 29 type 2 diabetes susceptible loci in the Caucasian population, including *KCNQ1* (rs2237895, rs231362), *CDKAL1* (rs7756992), *CDKN2BAS* (rs10811661), *TCF7L2* (rs7903146), *HHEX* (rs1111875), *TCF2* (rs7501939), *WFS1* (rs10010131), *CDC123/CAMK1D* (rs12779790), *MTNR1B* (rs10830963), *FTO* (rs8050136, rs9939609), *ADAMTS9* (rs4607103), *JAZF1* (rs864745), *TSPAN8/LGR5* (rs7961581), *TP53INP1* (rs896854), *PRC1* (rs8042680), *GCKR* (rs780094), *ZFAND6* (rs11634397), *BCL11A* (rs243021), *KLF14* (rs972283), *ZBED3* (rs4457053), *HNF1A* (rs7957197), *CENTD2* (rs1552224), *NOTCH2* (rs10923931), *CHCHD9* (rs13292136), *PPARG* (rs1801282), *THADA* (rs7578597), *SLC30A8* (rs13266634), *HMGA2* (rs1531343), *DUSP9* (rs5945326) [6]–[18]. Only the SNPs with Genotyping success rate >90% were investigated (rs231362, rs13266634, rs1531343 and rs5945326 were excluded from the study for their genotyping success rate was lower than 90%). The overall mean calling rate of 27 SNPs was 99.47%. The concordance rate based on 229 genotyping duplication was 100%.

### Statistical analyses

Hardy Weinberg equilibrium test was done for each SNP by χ^2^ test before further analysis. Difference in allele frequency was determined by χ^2^ test, with OR and 95% CI estimated.

The association study was performed assuming an additive model. Genotype distributions between case and control were compared using logistic regression adjusted for geographical region, age and gender. OR and 95% CI were calculated before and after adjustment for BMI. Quantitative characteristics of normoglycaemic or newly diagnosed type 2 diabetes individuals were analyzed by linear regression adjusted for geographical region, age and gender, while BMI was adjusted when appropriate (apart from anthropometric traits including height, weight, WC, hip circumference, waist-hip-ratio and BMI), and regression coefficients (*β*s) were determined. All non-Gaussian distributed quantitative traits were subjected to natural logarithm transformation to approximately approach normal distribution. The association studies were adjusted for multiple testing by a permutation step of 1,000 times using the PLINK analysis software. χ^2^ test and Cochran-Armitage test was used to analyze differences of risk allele frequency and genotype distribution between IFH and IPH.

In order to determine the joint effects of associated SNPs (IFH associated SNPs included *KCNQ1*, *CDKN2BAS*, *TCF7L2*, *FTO* (rs8050136), *GCKR*, except *FTO* (rs9939609) which was from the same linkage disequilibrium block with rs8050136; IPH associated SNPs included *KCNQ1*, *CDKAL1*, *TCF7L2*, *TP53INP1*, *PRC1*, *GCKR*) on the risk of IFH and IPH, we categorized the individuals based on risk alleles they carried, as previous reported [19]. The analysis included individuals for whom genotypes of involved SNPs were available (sample size for IFH SNPs was 291/767/4060 (IFH/IPH/Control); sample size for IPH SNPs was 293/771/4053). Considering the association results, we defined the G allele of rs780094 as a risk allele of IFH SNPs, while A allele was the risk allele for IPH SNPs. Individuals were grouped based on the amount of risk alleles they carried. For the joint study of IFH associated SNPs, individuals were divided into 5 groups (≤1, 2, 3, 4, ≥5), while there were 6 groups for IPH SNP analysis (≤3, 4, 5, 6, 7, ≥8). The group carrying fewest risk alleles was considered as reference (OR = 1). Effective sizes for every unit increase in the number of risk alleles on IFH or IPH were calculated after adjustment for geographical region, age and gender using a logistic regression model.

All statistical analyses were performed using either SAS for Windows (version 9.2; SAS Institute, Cary, NC) or PLINK software (v1.05). All *p* values <0.05 (two-tailed) were considered statistically significant.

Power calculations were performed using Quanto software (available at http://hydra.usc.edu/gxe/) and shown in Table S7. Power was calculated using the ORs reported in the original studies [6]–[18] as well as sample size and minor allele frequencies (MAF) in the present study. An overall diabetes risk of 9.7% [1] among Chinese was used in power analysis of newly diagnosed type 2 diabetes. In addition, for power calculation of IFH and IPH, overall IFH and IPH risk (1.65%, 4.56%) were calculated from 9.7% multiplying by the proportion of IFH and IPH in newly diagnosed type 2 diabetes (17%, 47%) [1].

## Results

### Association study of 27 SNPs and newly diagnosed type 2 diabetes in Han Chinese

All SNPs were within Hardy-Weinberg equilibrium in controls (*p*>0.05; Table S1). *TCF7L2*, *CDKN2BAS*, *CDKAL*1, *KCNQ1*, *HHEX*, *TCF2*, *CENTD2* and *TP53INP1* showed associations with type 2 diabetes (ORs ranged between 1.089–1.385, *p* value ranged between 4.112×10^−7^–0.0311; Table S2). Three of the eight SNPs remained significant after adjustment for multiple comparisons (*p* value ranged between 0.0020–0.0080; Table S2).

The association study of SNPs and type 2 diabetes related quantitative traits showed that subjects carrying the *CDKAL1* risk allele had higher plasma glucose 30 min in OGTT (*β* = 0.01669 per G allele for lnFPG; *p* = 0.0006). The correlation remained after multiple test correction (Empirical *p* = 0.0140). In cases of newly diagnosed type 2 diabetes, subjects carrying the *KCNQ1* risk allele had lower BMI (*β* = −0.01738 per for C allele for lnBMI; *p* = 0.0001), and this correlation remained after multiple test correction (Empirical *p* = 0.0070) (Table S3).

### Clinical characteristics of individuals with IFH and IPH

As shown in Table 1, individuals with IFH had higher FPG, liver insulin resistance (evaluated by HOMA-IR and ISIm) and better early phase insulin secretion ability (evaluated by ΔI_30_/ΔG_30_) compared to subjects with IPH. 2h PG of individuals with IPH was higher, as well as higher prevalence of cardiovascular disease. HOMA-B was lower in IFH than IPH, which was calculated from FPG and fasting insulin level.

### Association study of SNPs and IFH in Hans

Table 2 shows that *TCF7L2*, *CDKN2BAS*, *KCNQ1*, *FTO* and *GCKR* are significantly associated with IFH (OR ranged between 1.171–1.524; *p* value ranged between 0.0023–0.0476). When adjusted for region, age and gender, *TCF7L2* and *FTO* (rs8050136, rs9939609) risk alleles conferred 1.454-, 1.403- and 1.398-fold increased risk of IFH. After further adjustment for BMI, the significant association between IFH with *TCF7L2* or *CDKN2BAS* remained (rs7903146: 1.503 [1.023–2.208], *p* = 0.0379; rs10811661: 1.220 [1.034–1.441], *p* = 0.0188), while *KCNQ1* was no longer associated with IFH (1.194 [0.991–1.439], *p* = 0.0617). SNPs in *FTO* were not associated with IFH after adjustment for BMI (rs8050136: 1.263 [0.995–1.604], *p* = 0.0554; rs9939609: 1.253 [0.987–1.590], *p* = 0.0642) with a reduction in OR values.

**Table 2:** SNPs significantly associated with isolated fasting hyperglycemia in Hans.

		Minor/major		Allelic	Genotypic	Genotypic
Gene	SNP	allele^a^		association^b^	association^c^	association^d^
*TCF7L2*	rs7903146	**T**/C	OR (95%CI)	1.524 (1.074,2.164)	1.454 (1.019,2.075)	1.503 (1.023,2.208)
			*p*	**0.0176**	**0.0390**	**0.0379**
			Empirical *p*	0.4236		
*KCNQ1*	rs2237895	**C**/A	OR (95%CI)	1.201 (1.008,1.431)	1.211 (1.016,1.444)	1.194 (0.991,1.439)
			*p*	**0.0401**	**0.0327**	0.0617
			Empirical *p*	0.7003		
*CDKN2BAS*	rs10811661	C/**T**	OR (95%CI)	1.189 (1.016,1.392)	1.183 (1.011,1.384)	1.220 (1.034,1.441)
			*p*	**0.0310**	**0.0358**	**0.0188**
			Empirical *p*	0.6034		
*FTO*	rs8050136	**A**/C	OR (95%CI)	1.405 (1.129,1.750)	1.403 (1.125,1.750)	1.263 (0.995,1.604)
			*p*	**0.0023**	**0.0027**	0.0554
			Empirical *p*	0.0629		
*FTO*	rs9939609	**A**/T	OR (95%CI)	1.394 (1.119,1.735)	1.398 (1.120,1.744)	1.253 (0.987,1.590)
			*p*	**0.0029**	**0.0030**	0.0642
			Empirical *p*	0.0819		
*GCKR*	rs780094	**G**/A	OR (95%CI)	1.171 (1.002,1.369)	1.167 (0.999,1.362)	1.166 (0.989,1.374)
			*p*	**0.0476**	0.0513	0.0678
			Empirical *p*	0.7562		

a Risk alleles for type 2 diabetes in the Caucasian descent population are denoted in bold. OR and 95% CI are reported for the allele with higher type 2 diabetes risk previously reported for Caucasians using χ2 or an additive model in logistic regression.

b Comparison of the allelic distribution between isolated fasting hyperglycemia and controls.

c Comparison of the genotype distribution between isolated fasting hyperglycemia and controls after adjusting for region, age and gender.

d Comparison of the genotype distribution between isolated fasting hyperglycemia and controls after adjusting for region, age, gender and BMI.

Associations of the rest SNPs with isolated fasting hyperglycemia are shown in Table S4.

Empirical *p* values were calculated through 1,000 permutations. *p* values <0.05 are shown in bold.

**Figure 1:**
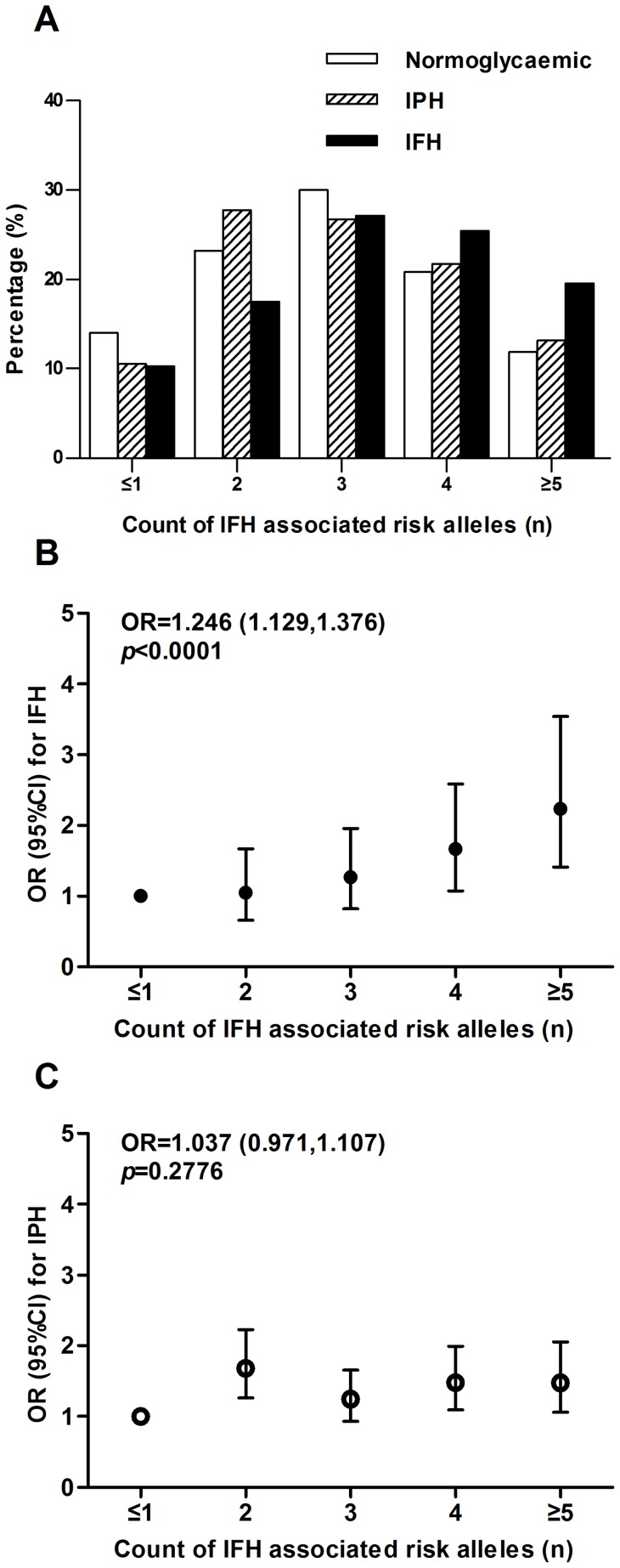
Combined effects of increasing numbers of isolated fasting hyperglycaemia-associated risk alleles for *TCF7L2*, *CDKN2BAS*, *KCNQ1*, *FTO* and *GCKR*. A: The isolated fasting hyperglycaemia-associated risk allele distribution in controls, participants with isolated fasting hyperglycaemia and isolated postprandial hyperglycaemia. Each additional risk allele increased the risk of isolated fasting hyperglycaemia by 1.24-fold (*p*<0.0001) (B) but not the risk of isolated postprandial hyperglycaemia (C). OR and 95% CI plotted on the y-axis for the corresponding number of risk alleles on the x-axis (black circles  =  risk of isolated fasting hyperglycaemia; white circles  =  risk of isolated postprandial hyperglycaemia). B: Participants harboring five or more risk alleles had a 2.23-fold increased risk for isolated fasting hyperglycaemia (*p*<0.0001) compared with the reference group. C: However, risk for isolated postprandial hyperglycaemia was not increased as risk allele number increased. IFH, isolated fasting hyperglycemia; IPH, isolated postprandial hyperglycemia.

Joint study of IFH associated SNPs, including *TCF7L2*, *CDKN2BAS*, *KCNQ1*, *FTO* and *GCKR*, shows that individuals with IFH carry more risk alleles from the above loci than controls (*p_trend_* <0.0001; Figure 1A). Risk of IFH increased 24.6% for each additional risk allele carried (1.246 [1.129–1.376] per allele, *p*<0.0001; Figure 1B). However, individuals with IPH did not carry more risk alleles than controls (*p_trend_*  = 0.2208; Figure 1A). The number of risk alleles did not increase the risk of IPH (1.037 [0.971–1.107] per allele, *p* = 0.2776; Figure 1C).

### Association study of SNPs and IPH in Han Chinese

Table 3 shows that *TCF7L2*, *CDKAL1*, *KCNQ1*, and *PRC1* are significantly associated with IPH (OR ranged between 1.154–1.709, *p* value ranged between 0.00038–0.03482). Another two SNPs, *TP53INP1* and *GCKR*, were also found to be associated with IPH after adjustment for region, age and gender (rs896854: 1.129 [1.009–1.263], *p* = 0.0348; rs780094: 0.891 [0.801–0.991], *p* = 0.0331). The *PRC1* and *TCF7L2* risk alleles conferred a 1.758- and 1.485-fold of increased risk of IPH, respectively. Risk alleles in *CDKAL1, KCNQ1* and *TP53INP1* conferred a 1.217-, 1.153- and 1.129-fold of increased risk of IPH, respectively. Noticeably, carriers of the validated type 2 diabetes risk allele G of *GCKR* showed lower risk of IPH; therefore the A allele of *GCKR* was defined as the risk allele in further joint study. *TCF7L2*, *CDKAL1* and *KCNQ1* remained associated with IPH after adjustment for BMI (rs7903146: 1.438 [1.080–1.888], *p* = 0.0123; rs7756992: 1.233 [1.095–1.389], *p* = 0.0005; rs2237895: 1.181 [1.038–1.343], *p* = 0.0115). *GCKR*, *TP53INP1* and *PRC1* were not associated with IPH after adjustment for BMI (rs780094: 0.895 [0.796–1.006], *p* = 0.0627; rs896854: 1.099 [0.971–1.245], *p* = 0.1360; rs8042680: 1.555 [0.917–2.639], *p* = 0.1016).

**Table 3:** SNPs significantly associated with isolated postprandial hyperglycemia in Hans.

		Minor/major		Allelic	Genotypic	Genotypic
Gene	SNP	allele^a^		association^b^	association^c^	association^d^
*TCF7L2*	rs7903146	**T**/C	OR (95%CI)	1.507 (1.187,1.914)	1.485 (1.158,1.906)	1.438 (1.080,1.888)
			*p*	**0.0007**	**0.0019**	**0.0123**
			Empirical *p*	**0.0230**		
*KCNQ1*	rs2237895	**C**/A	OR (95%CI)	1.154 (1.030,1.294)	1.153 (1.025,1.296)	1.181 (1.038,1.343)
			*p*	**0.0137**	**0.0174**	**0.0115**
			Empirical *p*	0.3317		
*GCKR*	rs780094	**G**/A	OR (95%CI)	0.904 (0.815,1.003)	0.891 (0.801,0.991)	0.895 (0.796,1.006)
			*p*	0.0574	**0.0331**	0.0627
			Empirical *p*	0.8182		
*CDKAL1*	rs7756992	A/**G**	OR (95%CI)	1.209 (1.089,1.342)	1.217 (1.092,1.355)	1.233 (1.095,1.389)
			*p*	**0.0004**	**0.0004**	**0.0005**
			Empirical *p*	**0.0180**		
*TP53INP1*	rs896854	**A**/G	OR (95%CI)	1.109 (0.996,1.235)	1.129 (1.009,1.263)	1.099 (0.971,1.245)
			*p*	0.0601	**0.0348**	0.1360
			Empirical *p*	0.8282		
*PRC1*	rs8042680	C/**A**	OR (95%CI)	1.709 (1.045,2.793)	1.758 (1.063,2.908)	1.555 (0.917,2.639)
			*p*	**0.0307**	**0.0279**	0.1016
			Empirical *p*	0.6164		
*HHEX*	rs1111875	**G**/A	OR (95%CI)	1.105 (0.987,1.238)	1.102 (0.981,1.238)	1.151 (1.013,1.309)
			*p*	0.0838	0.1027	**0.0309**
			Empirical *p*	1.0000		

a Risk alleles for type 2 diabetes in the Caucasian descent population are denoted in bold. OR and 95% CI are reported for the allele with higher type 2 diabetes risk previously reported for Caucasians using χ2 or an additive model in logistic regression.

b Comparison of the allelic distribution between isolated postprandial hyperglycemia and controls.

c Comparison of the genotype distribution between isolated postprandial hyperglycemia and controls after adjusting for region, age and gender.

d Comparison of the genotype distribution between isolated postprandial hyperglycemia and controls after adjusting for region, age, gender and BMI.

Associations of the rest SNPs with isolated postprandial hyperglycemia are shown in Table S5.

Empirical *p* values were calculated through 1,000 permutations. *p* values <0.05 are shown in bold.

### Difference of allelic frequencies and genotype distributions between IFH and IPH

Among all tested SNPs, risk allele frequencies of SNPs in *FTO* and *GCKR* in IFH were much higher than those in IPH (A allele of rs8050136, *p* = 0.0026; A allele of rs9939609, *p* = 0.0053; G allele of rs780094, *p* = 0.0043; Table 4). However risk allele frequencies of SNPs in *CDKAL1* were much lower in IFH subjects than IPH subjects (G allele of rs7756992, *p* = 0.0103). Genotype distributions of these SNPs between IFH and IPH were significantly different (rs7756992 *p_trend_*  = 0.0122; rs8050136 *p_trend_*  = 0.0024; rs9939609 *p_trend_*  = 0.0049; rs780094 *p_trend_*  = 0.0037). Allele frequencies or genotype distributions of *CDKN2BAS*, *TP53INP1* and *PRC1* were statistically comparable between IFH and IPH (Table S6).

**Table 4:** SNPs showed significant differences in risk allele frequency and genotype distribution between isolated fasting hyperglycemia and isolated postprandial hyperglycemia.

			Risk allele		χ^2^ test of risk		Genotype distribution		Cochran-Armitage trend test	
	Minor/major		frequency		allele frequency		(BB/Bb/bb)		of genotype distribution	
Gene	SNP	allele^a^	IFH	IPH	χ^2^	p	IFH	IPH	Z	Two-tails p
*FTO*	rs8050136	**A**/C	0.153	0.109	9.0651	**0.0026**	4/96/240	12/163/687	−3.0300	**0.0024**
*FTO*	rs9939609	**A**/T	0.153	0.111	7.7725	**0.0053**	4/96/241	12/168/684	−2.8116	**0.0049**
*GCKR*	rs780094	**G**/A	0.515	0.450	8.1464	**0.0043**	89/172/79	166/446/252	−2.8990	**0.0037**
*CDKAL1*	rs7756992	A/**G**	0.510	0.568	6.5872	**0.0103**	94/160/87	286/405/169	2.5059	**0.0122**

a Risk alleles for type 2 diabetes in Caucasians are denoted in bold.

Allelic frequencies between isolated fasting hyperglycemia and isolated postprandial hyperglycemia were compared using χ2 test.

Genotype distributions are shown as the counts of three genotypes (BB, Bb, bb). B, risk allele; b, non-risk allele. Genotype distributions between isolated fasting hyperglycemia and isolated postprandial hyperglycemia were compared using Cochran-Armitage trend test.

Comparisons of allele frequency and genotype distribution of the rest SNPs are shown in Table S6.

*p* values <0.05 are shown in bold.

IFH, isolated fasting hyperglycemia; IPH, isolated postprandial hyperglycemia.

## Discussion

Current understanding on the genetic etiology of type 2 diabetes has been greatly improved over past decades. Considering the high heterogeneity of IFH and IPH, we proposed the hypothesis that genetic bases of IFH and IPH are different. Further, we speculated that genetic variants in loci modulating insulin sensitivity promoted IFH, while variants in loci affecting beta cell function promoted IPH.

The present study indicated that gene variants from *FTO* and *GCKR* were specifically associated with increased risk of IFH, while gene variant from *CDKAL1* was associated with increased risk of IPH. It also showed that risk allele frequencies of *FTO* and *GCRK* in IFH were higher than that in IPH. However, risk allele frequency of *CDKAL1* in IFH was lower than that in IPH.

*FTO* was initially found to be associated with obesity in Caucasians [20]. In this study, we also confirmed the association of *FTO* with obesity (shown as WC and BMI) in newly diagnosed type 2 diabetes. It was reported that the fat mass and obesity associated protein, which is the genetic product of *FTO,* plays a role in the regulation of insulin sensitivity and insulin secretion from pancreatic beta cells [21]. Berulava *et al.* [22] found that SNP rs9939609 influenced the transcription of *FTO*. *FTO* SNPs rs8050136 and rs9939609, located in the same linkage disequilibrium block, were first found to be associated with type 2 diabetes in Caucasians [11], [12]. According to Hapmap, risk allele (A) frequency of rs8050136 and rs9939609 are much higher in Caucasians (0.450), compared to East Asians (0.144). Studies in the Han Chinese population failed to establish consistency of association between *FTO* variants and type 2 diabetes [23]–[28]. Further, no association remained between *FTO* and type 2 diabetes after BMI adjustment in some studies, indicating that *FTO* variants were associated with increased risk of type 2 diabetes through modulating the process of obesity development [25]–[27]. However, some reports do not support this viewpoint [23], [24]. It had been well accepted that these contradictory findings result from the low MAF in Han Chinese, which results in insufficient statistical power [23], [26], [28].

In the present study, we found that SNPs in *FTO* are associated with a 40% increased risk for IFH. Furthermore, we found OR decreased to 25–26% after adjustment for BMI (*p*>0.05, as the sample size of IFH was not sufficient large). On the other hand, SNPs in *FTO* were not associated with IPH (Table S5). We postulate that risk alleles of SNPs in *FTO* (rs8050136, rs9939609) or causal variants genetically linked with them may specifically promote risk of IFH in Han Chinese, which is partially independent of general obesity (evaluated by BMI).

Based on these observations, we speculate that previous findings of the association between *FTO* and type 2 diabetes may have resulted from the different proportions of IFH and IPH in those type 2 diabetes populations. According to DECODE and DECODA, the ratios of IFH/IPH were 613/473 (40.4%/28.4%) in Caucasians and 220/546 (18.1%/44.9%) in Asians [3], [29]. In the CNDMDS performed in 2007–2008, the ratio of IFH/IPH was 17%/47% (unpublished data) in Han Chinese. Previous studies have demonstrated that insulin resistance in Caucasians is much higher than Han Chinese, while Han Chinese typically have worse beta cell function than Caucasians [3], [29]. Therefore, *FTO* is well established as a susceptibility gene of type 2 diabetes in Caucasians [11], [12], but not in Chinese. No association between *FTO* and type 2 diabetes was observed in our population of newly diagnosed patients.

Glucokinase regulatory protein is the product of *GCKR* genetic encoding, whose overexpression could ameliorate insulin sensitivity and glucose tolerance in mice, while leading to higher serum TG [30]. *GCKR* gene polymorphisms were initially reported to be associated with TG levels [15]. In the present study, we confirm that the G allele of rs780094 in *GCKR* is associated with increased FPG and lower TG levels in newly diagnosed type 2 diabetes of Han ancestry (Table S3).

*GCKR* (rs780094) was first found to be associated with type 2 diabetes in Caucasians [15]. A nonsynonymous variant of *GCKR* (rs1260326, P446L) has strong linkage disequilibrium with rs780094 according to HapMap II data (CEU: r^2^ = 0.93; CHB/JPT: r^2^ = 0.83). This variant could regulate insulin secretion and blood TG levels through regulating the activity of glucokinase in the liver, while it was also correlated with type 2 diabetes [31]. In Han Chinese, the association of rs780094 with type 2 diabetes did not allow for consistent conclusions to be drawn [25], [32]–[35]. Notably, we found that the G allele was associated with increased risk of IFH, but decreased risk of IPH. Therefore, it's not surprising that we cannot confirm a correlation between rs780094 and newly diagnosed type 2 diabetes. We suppose that risk alleles of causal variants at the same linkage disequilibrium region with rs780094 specifically promote the progression of IFH. Therefore, the proportion of IFH and IPH adds to the debate over whether *GCKR* is a susceptibility gene for type 2 diabetes in Han Chinese.

Genetic product of the *CDKAL1* gene belongs to the mammalian methylthyiotransferase family, and is involved in the process of synthesis and cleavage of proinsulin by ensuring the accurate translation of Lys codons [36]. It has been reported that the proinsulin/insulin conversion rate of *CDKAL1* (rs7754840) risk allele carriers were lower [37]. Variations in *CDKAL1* have been reported to be associated with impaired insulin secretion and the increased risk of type 2 diabetes. The association of *CDKAL1* (rs7756992) with type 2 diabetes has been well established in Han Chinese [19], [38], [39]. In this study, we found that G allele of rs7756992 was associated with increased risk of IPH, rather than IFH (Table S4). Though the sample size of IFH cases was smaller than that of IPH, allele frequencies and genotype distribution of rs7756992 were significantly different and the power to detect association of IFH and rs7756992 reached 79.49% (Table S7). Moreover, it was reported that rs7756992, a common variant in *CDKAL1* loci (MAF  = 0.479), contributed to type 2 diabetes risk in Han Chinese in a study with a similar sample size [39]. Therefore, we speculate that the risk allele of rs7756992 was specifically associated with increased risk of IPH. Association of rs7756992 with newly diagnosed type 2 diabetes was also confirmed in present study, which likely resulted from the high proportion of IPH in the Han Chinese population.

We also observed that IFH and IPH share common susceptible SNPs including *TCF7L2* and *KCNQ1*, both of which played approximately equal effect sizes in IFH and IPH subjects, respectively. *TCF7L2* and *KCNQ1* were previously considered to be the strongest type 2 diabetes susceptible genes in Caucansians and East Asians [9], [12]–[15], [17]–[20], [40]–[43], both of which were well replicated in Han Chinese [25], [35], [40], [41], [44], [45]. Both *TCF7L2* and *KCNQ1* have been shown to play essential roles in beta cell survival and function, and they have been suggested to be involved in the modulation of insulin sensitivity and obesity [42], [43], [46]–[49]. Based on our observations, causal variants in linkage disequilibrium with rs7903146 and rs2237895 were the common hereditary bases of IFH and IPH, resulting in their similar clinical features. In addition, Yu, *et al.* demonstrated that the C allele of rs2237895 showed a greater effect on diabetes risk in participants with lower BMI, as well as a correlation between BMI and rs2237895 [50]. Our results, and other studies, support the idea that subjects carrying risk alleles at rs2237895 have lower BMI in newly diagnosed type 2 diabetes patients.

Though we confirm that *CDKN2BAS* is associated with IFH, while *PRC1* and *TP53INP1* are associated with IPH, the allele frequencies of the three SNPs were not found to be different between IFH and IPH (Table S6). However, it should not be ignored that only 341 IFH individuals were recruited, resulting in a lower or insufficient statistical power for IFH (Table S7). Further investigation of large samples at these loci is warranted to elucidate their association with IPH or IFH.

We observed that the five variants associated with IFH were additively associated with the increased risk of IFH. Moreover, six variants associated with IPH were additively associated with the increased risk of IPH. Despite of the common hereditary bases, different joint effects of SNPs on IFH and IPH groups also reflect hereditary differences.

The present study has some limitations to be addressed. First, the study could not achieve enough statistical power for several SNPs because of the limited sample size (in particular, the sample size of incident IFH was relatively small). Moreover, all SNPs involved in the study have been validated to be type 2 diabetes susceptible SNPs previously; thus novel loci could not be established by our work. However, this is the first study to investigate the susceptibility genes of IFH and IPH based on a general population sample which gave us samples of IFH and IPH. It also provides clues regarding targeted regions for future genetic research on IFH and IPH.

In conclusion, our study indicates that both distinct (*FTO, GCKR*, *CDKAL1*) and common genetic bases (*TCF7L2*, *KCNQ1*) exist for IPH and IFH in Han Chinese, suggesting specific underlying pathogenic mechanisms resulting in the heterogeneity of their clinic manifestation. Given the differences in risk allele frequencies, it will be valuable to further examine these genes thoroughly to search for the culprit disease loci of IPH and IFH. It also provides strong evidence for prediction, prevention, diagnosis, personalized medicine and drug development for Han Chinese with type 2 diabetes, among which most of the newly diagnosed type 2 diabetes patients have IPH.

## Supporting Information

Table S1 Risk allele frequency, genotype distribution and Hardy-Weinberg equilibrium in study populations. ^a^ Risk alleles for type 2 diabetes in Caucasians are denoted in bold. Genotype distributions are shown as the counts of three genotypes (BB, Bb, bb). B, risk allele; b, non-risk allele. *p*_HWE_ <0.05 is shown in bold, suggesting the SNP was not in Hardy-Weinberg equilibrium in the given population. IFH, isolated fasting hyperglycemia; IPH, isolated postprandial hyperglycemia.
(DOC)

Table S2 Association between SNPs and newly diagnosed type 2 diabetes in Han Chinese. ^a^ Risk alleles for type 2 diabetes in Caucasians are denoted in bold. OR and 95% CI are reported for the allele with higher type 2 diabetes risk as previously reported in Caucasians using χ^2^ or an additive model in logistic regression. ^b^ Comparison of the allelic distribution between type 2 diabetes and controls. ^c^ Comparison of the genotype distribution between type 2 diabetes and controls after adjusting for region, age and gender. ^d^ Comparison of the genotype distribution between type 2 diabetes and controls after adjusting for region, age, gender and BMI. Empirical *p* values were calculated through 1,000 permutations. *p* values<0.05 are shown in bold.
(DOC)

Table S3 Association between SNPs and clinical features in control and newly diagnosed type 2 diabetes subjects. *β* value is reported for the allele with higher type 2 diabetes risk as previously reported in Caucasians using an additive model in multi-variate linear regression adjusted for region, gender and age, with BMI when appropriate. Associations with *p* value<0.05 are shown in the table. Empirical *p* values were calculated through 1,000 permutations. Empirical *p* values<0.05 are shown in bold.
(DOC)

Table S4 SNPs did not show significant association with isolated fasting hyperglycemia in Hans. ^a^ Risk alleles for type 2 diabetes in the Caucasian descent population are denoted in bold. OR and 95% CI are reported for the allele with higher type 2 diabetes risk previously reported for Caucasians using χ2 or an additive model in logistic regression. ^b^ Comparison of the allelic distribution between isolated fasting hyperglycemia and controls. ^c^ Comparison of the genotype distribution between isolated fasting hyperglycemia and controls after adjusting for region, age and gender. ^d^ Comparison of the genotype distribution between isolated fasting hyperglycemia and controls after adjusting for region, age, gender and BMI. We failed to compare genotype distribution between isolated fasting hyperglycemia and controls at rs7957197 because its minor allele frequency of present samples was very low. Empirical *p* values were calculated through 1,000 permutations. *p* values<0.05 are shown in bold.
(DOC)

Table S5 SNPs did not show significant association with isolated postprandial hyperglycemia in Hans. ^a^ Risk alleles for type 2 diabetes in the Caucasian descent population are denoted in bold. OR and 95% CI are reported for the allele with higher type 2 diabetes risk previously reported for Caucasians using χ2 or an additive model in logistic regression. ^b^ Comparison of the allelic distribution between isolated postprandial hyperglycemia and controls. ^c^ Comparison of the genotype distribution between isolated postprandial hyperglycemia and controls after adjusting for region, age and gender. ^d^ Comparison of the genotype distribution between isolated postprandial hyperglycemia and controls after adjusting for region, age, gender and BMI. Empirical *p* values were calculated through 1,000 permutations. *p* values <0.05 are shown in bold.
(DOC)

Table S6 SNPs did not show significant differences in risk allele frequency or genotype distribution between isolated fasting hyperglycemia and isolated postprandial hyperglycemia. ^a^ Risk alleles for type 2 diabetes in Caucasians are denoted in bold. Allelic frequencies between isolated fasting hyperglycemia and isolated postprandial hyperglycemia were compared using χ2 test. Genotype distributions are shown as the counts of three genotypes (BB, Bb, bb). B, risk allele; b, non-risk allele. Genotype distributions between isolated fasting hyperglycemia and isolated postprandial hyperglycemia were compared using Cochran-Armitage trend test. *p* values <0.05 are shown in bold. IFH, isolated fasting hyperglycemia; IPH, isolated postprandial hyperglycemia.
(DOC)

Table S7 Power of the association studies. ^a^ Risk alleles for type 2 diabetes in Caucasians are denoted in bold. ^b^ ORs and 95% CIs reported by reference studies (Ref.). ^c^ Power to detect the association between SNPs and newly diagnosed type 2 diabetes was estimated under the additive model and given the reported OR(95%CI), sample size and α = 0.05 (two-sided). ^d^ Power to detect the association between SNPs and isolated fasting hyperglycaemia was estimated under the additive model and given the reported OR(95%CI), sample size and α = 0.05 (two-sided). ^e^ Power to detect the association between SNPs and isolated postprandial hyperglycaemia was estimated under the additive model and given the reported OR(95%CI), sample size and α = 0.05 (two-sided). IFH, isolated fasting hyperglycemia; IPH, isolated postprandial hyperglycemia.
(DOC)
