# Correction: Association of Genetic Variants with Isolated Fasting Hyperglycaemia and Isolated Postprandial Hyperglycaemia in a Han Chinese Population

**DOI:** 10.1371/annotation/936a4359-1bf5-4c33-be7d-1468e75eaa8b

**Published:** 2013-08-22

**Authors:** Xiaomu Kong, Jing Hong, Ying Chen, Li Chen, Zhigang Zhao, Qiang Li, Jiapu Ge, Gang Chen, Xiaohui Guo, Juming Lu, Jianping Weng, Weiping Jia, Linong Ji, Jianzhong Xiao, Zhongyan Shan, Jie Liu, Haoming Tian, Qiuhe Ji, Dalong Zhu, Zhiguang Zhou, Guangliang Shan, Wenying Yang

In the PDF, in the Results section, the second paragraph of subsection "Association study of SNPs and IPH in Han Chinese" was omitted. The paragraph is available below:

Joint study of IPH associated SNPs, including *TCF7L2*, *CDKAL1*, *KCNQ1*, *PRC1*, *TP53INP1* and *GCKR*, shows that individuals with IPH carry more risk alleles from the above loci than controls (*p_trend_* < 0.0001; Figure 2A). Risk of IPH increased 19.0% for each additional risk allele carried (1.190 [1.120-1.263] per allele, *p* < 0.0001; Figure 2B). In SNPs associated with IPH, the numbers of risk alleles carried in IFH subjects were not significantly more than that in the control group (*p_trend_* = 0.2752; Figure 2A); and the number of risk alleles did not increase the risk of IFH (1.053 [0.963-1.150] per allele, p = 0.2564; Figure 2C). 

