# Supplementary material for: Association of Genetic Variants with Isolated Fasting Hyperglycaemia and Isolated Postprandial Hyperglycaemia in a Han Chinese Population
Source: PLoS One. 2013 Aug 19;8(8):e71399. doi: 10.1371/journal.pone.0071399 (PMC3747192; doi:10.1371/journal.pone.0071399)
Supplement: Table S1 — Risk allele frequency, genotype distribution and Hardy-Weinberg equilibrium in study populations. a Risk alleles for type 2 diabetes in Caucasians are denoted in bold. Genotype distributions are shown as the counts of three genotypes (BB, Bb, bb). B, risk allele; b, non-risk allele. p HWE <0.05 is shown in bold, suggesting the SNP was not in Hardy-Weinberg equilibrium in the given population. IFH, isolated fasting hyperglycemia; IPH, isolated postprandial hyperglycemia. (DOC) [file pone.0071399.s001.doc]

**Supplementary Tables**

**Table S1.** Risk allele frequency, genotype distribution and Hardy-Weinberg equilibrium in study populations.

|  |  | **Chromosome** | | **Minor/** |  | | | **Genotype distribution** | | |  |  |  |
| --- | --- | --- | --- | --- | --- | --- | --- | --- | --- | --- | --- | --- | --- |
|  |  |  | **Position** | **major** | **Risk allele frequency** | | | **(BB/Bb/bb)** | | | ***p*_HWE_** | | |
| **Gene** | **SNP** | **Chr.** | **(Build 36.3)** | **allele^a^** | **Control** | **IFH** | **IPH** | **Control** | **IFH** | **IPH** | **Control** | **IFH** | **IPH** |
| *TCF7L2* | rs7903146 | 10 | 108385604 | **T**/C | 0.036 | 0.054 | 0.054 | 4/299/3929 | 1/35/305 | 2/88/767 | 0.660 | 1.000 | 1.000 |
| *KCNQ1* | rs2237895 | 11 | 2645543 | **C**/A | 0.316 | 0.357 | 0.348 | 416/1766/1925 | 32/146/116 | 100/343/337 | 0.718 | 0.204 | 0.386 |
| *CDKN2BAS* | rs10811661 | 9 | 22097368 | C/**T** | 0.526 | 0.569 | 0.542 | 1180/2100/958 | 118/151/71 | 241/453/168 | 0.689 | 0.097 | 0.099 |
| *FTO* | rs8050136 | 16 | 39703105 | **A**/C | 0.114 | 0.153 | 0.109 | 63/841/3343 | 4/96/240 | 12/163/687 | 0.224 | 0.140 | 0.482 |
| *FTO* | rs9939609 | 16 | 39707357 | **A**/T | 0.114 | 0.153 | 0.111 | 62/846/3334 | 4/96/241 | 12/168/684 | 0.324 | 0.140 | 0.606 |
| *GCKR* | rs780094 | 2 | 27483120 | **G**/A | 0.475 | 0.515 | 0.450 | 988/2059/1198 | 89/172/79 | 166/446/252 | 0.074 | 0.914 | 0.217 |
| *CDKAL1* | rs7756992 | 6 | 20623678 | A/**G** | 0.521 | 0.510 | 0.568 | 1134/2149/956 | 94/160/87 | 286/405/169 | 0.325 | 0.279 | 0.238 |
| *TP53INP1* | rs896854 | 8 | 91168409 | **A**/G | 0.343 | 0.368 | 0.367 | 473/1967/1805 | 40/171/130 | 118/397/348 | 0.071 | 0.164 | 0.770 |
| *PRC1* | rs8042680 | 15 | 67632326 | C/**A** | 0.982 | 0.988 | 0.990 | 4101/148/1 | 333/8/0 | 847/18/0 | 1.000 | 1.000 | 1.000 |
| *HHEX* | rs1111875 | 10 | 88089440 | **G**/A | 0.282 | 0.299 | 0.302 | 344/1695/2193 | 29/145/166 | 80/358/419 | 0.518 | 0.797 | 0.808 |
| *TCF2* | rs7501939 | 17 | 32038720 | **T**/C | 0.265 | 0.282 | 0.284 | 304/1639/2297 | 34/124/183 | 78/336/451 | 0.636 | 0.062 | 0.182 |
| *WFS1* | rs10010131 | 4 | 6226670 | A/**G** | 0.954 | 0.959 | 0.956 | 3867/376/7 | 313/28/0 | 791/70/3 | 0.601 | 1.000 | 0.225 |
| *CDC123/CAMK1D* | rs12779790 | 10 | 12244629 | **G**/A | 0.164 | 0.166 | 0.165 | 117/1156/2963 | 11/91/238 | 29/226/606 | 0.737 | 0.556 | 0.173 |
| *MTNRIB* | rs10830963 | 11 | 88799685 | **G**/C | 0.410 | 0.431 | 0.425 | 705/2058/1463 | 49/194/96 | 166/399/296 | 0.703 | **0.003** | 0.143 |
| *TSPAN8/LGR5* | rs7961581 | 12 | 68712783 | **C**/T | 0.201 | 0.215 | 0.189 | 165/1377/2698 | 11/124/205 | 28/269/564 | 0.535 | 0.150 | 0.656 |
| *THADA* | rs7578597 | 2 | 43468323 | C/**T** | 0.993 | 0.990 | 0.990 | 4183/58/0 | 333/7/0 | 841/17/0 | 1.000 | 1.000 | 1.000 |
| *JAZF1* | rs864745 | 7 | 28231122 | G/**A** | 0.764 | 0.741 | 0.748 | 2469/1518/240 | 185/131/22 | 478/328/52 | 0.733 | 1.000 | 0.717 |
| *PPARG* | rs1801282 | 3 | 12326481 | G/**C** | 0.936 | 0.938 | 0.935 | 3717/513/16 | 299/40/1 | 753/103/4 | 0.799 | 1.000 | 0.775 |
| *ADAMTS9* | rs4607103 | 3 | 64809108 | T/**C** | 0.625 | 0.625 | 0.636 | 1639/2017/578 | 133/160/48 | 351/395/116 | 0.294 | 1.000 | 0.769 |
| *NOTCH2* | rs10923931 | 1 | 118376489 | **T**/G | 0.034 | 0.031 | 0.038 | 7/279/3961 | 0/21/319 | 3/60/800 | 0.351 | 1.000 | 0.123 |
| *BCL11A* | rs243021 | 2 | 60326591 | C/**T** | 0.682 | 0.675 | 0.677 | 1969/1849/425 | 157/146/38 | 401/369/95 | 0.777 | 0.624 | 0.484 |
| *ZBED3* | rs4457053 | 5 | 71631811 | **G**/A | 0.050 | 0.053 | 0.058 | 8/407/3801 | 1/34/304 | 4/92/760 | 0.517 | 1.000 | 0.525 |
| *KLF14* | rs972283 | 7 | 124782393 | A/**G** | 0.723 | 0.730 | 0.715 | 2236/1648/347 | 183/123/29 | 446/340/75 | 0.077 | 0.213 | 0.402 |
| *CHCHD9* | rs13292136 | 9 | 51784164 | T/**C** | 0.905 | 0.921 | 0.900 | 3480/733/35 | 287/52/1 | 699/154/9 | 0.654 | 0.709 | 0.849 |
| *CENTD2* | rs1552224 | 11 | 68728355 | G/**T** | 0.910 | 0.921 | 0.919 | 3515/692/38 | 290/48/3 | 727/134/3 | 0.514 | 0.454 | 0.356 |
| *HNF1A* | rs7957197 | 12 | 118469299 | A/**T** | 0.998 | 1.000 | 0.998 | 4218/17/0 | 341/0/0 | 862/3/0 | 1.000 | 1.000 | 1.000 |
| *ZFAND6* | rs11634397 | 15 | 57190264 | **G**/A | 0.096 | 0.079 | 0.101 | 34/746/3461 | 2/50/289 | 12/150/701 | 0.425 | 1.000 | 0.256 |

^a^ Risk alleles for type 2 diabetes in Caucasians are denoted in bold.

Genotype distributions are shown as the counts of three genotypes (BB, Bb, bb). B, risk allele; b, non-risk allele.

*p*_HWE_ <0.05 is shown in bold, suggesting the SNP was not in Hardy-Weinberg equilibrium in the given population.

IFH, isolated fasting hyperglycemia; IPH, isolated postprandial hyperglycemia.
