# Supplementary material for: Association of Genetic Variants with Isolated Fasting Hyperglycaemia and Isolated Postprandial Hyperglycaemia in a Han Chinese Population
Source: PLoS One. 2013 Aug 19;8(8):e71399. doi: 10.1371/journal.pone.0071399 (PMC3747192; doi:10.1371/journal.pone.0071399)
Supplement: Table S2 — Association between SNPs and newly diagnosed type 2 diabetes in Han Chinese. a Risk alleles for type 2 diabetes in Caucasians are denoted in bold. OR and 95% CI are reported for the allele with higher type 2 diabetes risk as previously reported in Caucasians using χ2 or an additive model in logistic regression. b Comparison of the allelic distribution between type 2 diabetes and controls. c Comparison of the genotype distribution between type 2 diabetes and controls after adjusting for region, age and gender. d Comparison of the genotype distribution between type 2 diabetes and controls after adjusting for region, age, gender and BMI. Empirical p values were calculated through 1,000 permutations. p values<0.05 are shown in bold. (DOC) [file pone.0071399.s002.doc]

**Table S2**. Association between SNPs and newly diagnosed type 2 diabetes in Han Chinese.

|  |  | **Minor/major** | | **Allelic** | **Genotypic** | **Genotypic** |
| --- | --- | --- | --- | --- | --- | --- |
| **Gene** | **SNP** | **allele^a^** |  | **association^b^** | **association^c^** | **association^d^** |
| *TCF7L2* | rs7903146 | **T**/C | OR (95%CI) | 1.385 (1.165,1.647) | 1.340 (1.119,1.604) | 1.365 (1.113,1.675) |
|  |  |  | *p* | **0.0002** | **0.0014** | **0.0028** |
|  |  |  | Emprical *p* | **0.0040** |  |  |
| *KCNQ1* | rs2237895 | **C**/A | OR (95%CI) | 1.223 (1.131,1.322) | 1.228 (1.133,1.332) | 1.286 (1.174,1.409) |
|  |  |  | *p* | **0.0000** | **0.0000** | **0.0000** |
|  |  |  | Emprical *p* | **0.0020** |  |  |
| *CDKN2BAS* | rs10811661 | C/**T** | OR (95%CI) | 1.116 (1.040,1.198) | 1.122 (1.043,1.208) | 1.144 (1.052,1.243) |
|  |  |  | *p* | **0.0024** | **0.0021** | **0.0015** |
|  |  |  | Emprical *p* | 0.0639 |  |  |
| *FTO* | rs8050136 | **A**/C | OR (95%CI) | 1.107 (0.993,1.234) | 1.094 (0.979,1.222) | 1.003 (0.884,1.138) |
|  |  |  | *p* | 0.0661 | 0.1148 | 0.9651 |
|  |  |  | Emprical *p* | 0.8781 |  |  |
| *FTO* | rs9939609 | **A**/T | OR (95%CI) | 1.112 (0.998,1.240) | 1.105 (0.988,1.234) | 1.011 (0.891,1.146) |
|  |  |  | *p* | 0.0539 | 0.0794 | 0.8665 |
|  |  |  | Emprical *p* | 0.7982 |  |  |
| *GCKR* | rs780094 | **G**/A | OR (95%CI) | 1.036 (0.965,1.112) | 1.027 (0.955,1.105) | 1.034 (0.953,1.122) |
|  |  |  | *p* | 0.3335 | 0.4676 | 0.4239 |
|  |  |  | Emprical *p* | 1.0000 |  |  |
| *CDKAL1* | rs7756992 | A/**G** | OR (95%CI) | 1.138 (1.060,1.222) | 1.150 (1.069,1.238) | 1.181 (1.087,1.283) |
|  |  |  | *p* | **0.0004** | **0.0002** | **0.0001** |
|  |  |  | Emprical *p* | **0.0080** |  |  |
| *TP53INP1* | rs896854 | **A**/G | OR (95%CI) | 1.089 (1.012,1.173) | 1.099 (1.017,1.188) | 1.106 (1.013,1.208) |
|  |  |  | *p* | **0.0231** | **0.0171** | **0.0250** |
|  |  |  | Emprical *p* | 0.4805 |  |  |
| *PRC1* | rs8042680 | C/**A** | OR (95%CI) | 1.313 (0.979,1.761) | 1.350 (0.998,1.826) | 1.422 (1.012,1.999) |
|  |  |  | *p* | 0.0679 | 0.0518 | **0.0426** |
|  |  |  | Emprical *p* | 0.8901 |  |  |
| *HHEX* | rs1111875 | **G**/A | OR (95%CI) | 1.100 (1.018,1.189) | 1.108 (1.022,1.200) | 1.131 (1.033,1.238) |
|  |  |  | *p* | **0.0160** | **0.0126** | **0.0080** |
|  |  |  | Emprical *p* | 0.3806 |  |  |
| *TCF2* | rs7501939 | **T**/C | OR (95%CI) | 1.114 (1.030,1.206) | 1.133 (1.045,1.228) | 1.137 (1.038,1.245) |
|  |  |  | *p* | **0.0073** | **0.0025** | **0.0059** |
|  |  |  | Emprical *p* | 0.1878 |  |  |
| *WFS1* | rs10010131 | A/**G** | OR (95%CI) | 1.177 (0.985,1.405) | 1.212 (1.010,1.454) | 1.257 (1.024,1.544) |
|  |  |  | *p* | 0.0719 | **0.0391** | **0.0288** |
|  |  |  | Emprical *p* | 0.9021 |  |  |
| *CDC123/CAMK1D* | rs12779790 | **G**/A | OR (95%CI) | 1.083 (0.986,1.190) | 1.073 (0.975,1.181) | 1.116 (1.001,1.245) |
|  |  |  | *p* | 0.0971 | 0.1502 | **0.0470** |
|  |  |  | Emprical *p* | 0.9441 |  |  |
| *MTNRIB* | rs10830963 | **G**/C | OR (95%CI) | 1.068 (0.994,1.147) | 1.068 (0.991,1.15) | 1.068 (0.983,1.161) |
|  |  |  | *p* | 0.0731 | 0.0834 | 0.1204 |
|  |  |  | Emprical *p* | 0.9061 |  |  |
| *TSPAN8/LGR5* | rs7961581 | **C**/T | OR (95%CI) | 1.029 (0.943,1.124) | 1.042 (0.951,1.142) | 1.004 (0.906,1.113) |
|  |  |  | *p* | 0.5213 | 0.3751 | 0.9341 |
|  |  |  | Emprical *p* | 1.0000 |  |  |
| *THADA* | rs7578597 | C/**T** | OR (95%CI) | 0.743 (0.502,1.102) | 0.699 (0.465,1.051) | 0.690 (0.436,1.092) |
|  |  |  | *p* | 0.1380 | 0.0855 | 0.1131 |
|  |  |  | Emprical *p* | 0.9860 |  |  |
| *JAZF1* | rs864745 | G/**A** | OR (95%CI) | 0.957 (0.881,1.040) | 0.954 (0.876,1.040) | 0.998 (0.907,1.100) |
|  |  |  | *p* | 0.2989 | 0.2851 | 0.9747 |
|  |  |  | Emprical *p* | 1.0000 |  |  |
| *PPARG* | rs1801282 | G/**C** | OR (95%CI) | 1.004 (0.869,1.160) | 0.996 (0.857,1.157) | 1.000 (0.843,1.186) |
|  |  |  | *p* | 0.9604 | 0.9557 | 0.9989 |
|  |  |  | Emprical *p* | 1.0000 |  |  |
| *ADAMTS9* | rs4607103 | T/**C** | OR (95%CI) | 1.011 (0.940,1.088) | 1.006 (0.933,1.086) | 1.049 (0.963,1.143) |
|  |  |  | *p* | 0.7599 | 0.8677 | 0.2727 |
|  |  |  | Emprical *p* | 1.0000 |  |  |
| *NOTCH2* | rs10923931 | **T**/G | OR (95%CI) | 0.990 (0.815,1.202) | 1.012 (0.831,1.232) | 1.000 (0.799,1.251) |
|  |  |  | *p* | 0.9171 | 0.9084 | 0.9997 |
|  |  |  | Emprical *p* | 1.0000 |  |  |
| *BCL11A* | rs243021 | C/**T** | OR (95%CI) | 1.006 (0.932,1.085) | 1.005 (0.929,1.087) | 0.990 (0.906,1.082) |
|  |  |  | *p* | 0.8799 | 0.8958 | 0.8264 |
|  |  |  | Emprical *p* | 1.0000 |  |  |
| *ZBED3* | rs4457053 | **G**/A | OR (95%CI) | 0.964 (0.818,1.136) | 0.942 (0.794,1.116) | 0.950 (0.784,1.151) |
|  |  |  | *p* | 0.6591 | 0.4862 | 0.6001 |
|  |  |  | Emprical *p* | 1.0000 |  |  |
| *KLF14* | rs972283 | A/**G** | OR (95%CI) | 1.015 (0.938,1.099) | 1.017 (0.939,1.103) | 1.035 (0.945,1.134) |
|  |  |  | *p* | 0.7083 | 0.6762 | 0.4564 |
|  |  |  | Emprical *p* | 1.0000 |  |  |
| *CHCHD9* | rs13292136 | T/**C** | OR (95%CI) | 1.009 (0.894,1.138) | 1.002 (0.885,1.134) | 0.976 (0.849,1.123) |
|  |  |  | *p* | 0.8882 | 0.9801 | 0.7381 |
|  |  |  | Emprical *p* | 1.0000 |  |  |
| *CENTD2* | rs1552224 | G/**T** | OR (95%CI) | 1.151 (1.013,1.308) | 1.140 (0.999,1.302) | 1.073 (0.924,1.246) |
|  |  |  | *p* | **0.0311** | 0.0522 | 0.3543 |
|  |  |  | Emprical *p* | 0.5854 |  |  |
| *HNF1A* | rs7957197 | A/**T** | OR (95%CI) | 1.138 (0.533,2.432) | 1.032 (0.473,2.252) | 1.452 (0.585,3.606) |
|  |  |  | *p* | 0.7379 | 0.9363 | 0.4219 |
|  |  |  | Emprical *p* | 1.0000 |  |  |
| *ZFAND6* | rs11634397 | **G**/A | OR (95%CI) | 0.992 (0.879,1.119) | 0.983 (0.868,1.113) | 0.992 (0.861,1.143) |
|  |  |  | *p* | 0.8936 | 0.7857 | 0.9088 |
|  |  |  | Emprical *p* | 1.0000 |  |  |

^a^ Risk alleles for type 2 diabetes in Caucasians are denoted in bold.

OR and 95% CI are reported for the allele with higher type 2 diabetes risk as previously reported in Caucasians using χ^2^ or an additive model in logistic regression.

^b^ Comparison of the allelic distribution between type 2 diabetes and controls.

^c^ Comparison of the genotype distribution between type 2 diabetes and controls after adjusting for region, age and gender.

^d^ Comparison of the genotype distribution between type 2 diabetes and controls after adjusting for region, age, gender and BMI.

Empirical *p* values were calculated through 1,000 permutations. *p* values < 0.05 are shown in bold.
