# Supplementary material for: Association of Genetic Variants with Isolated Fasting Hyperglycaemia and Isolated Postprandial Hyperglycaemia in a Han Chinese Population
Source: PLoS One. 2013 Aug 19;8(8):e71399. doi: 10.1371/journal.pone.0071399 (PMC3747192; doi:10.1371/journal.pone.0071399)
Supplement: Table S3 — Association between SNPs and clinical features in control and newly diagnosed type 2 diabetes subjects. β value is reported for the allele with higher type 2 diabetes risk as previously reported in Caucasians using an additive model in multi-variate linear regression adjusted for region, gender and age, with BMI when appropriate. Associations with p value<0.05 are shown in the table. Empirical p values were calculated through 1,000 permutations. Empirical p values<0.05 are shown in bold. (DOC) [file pone.0071399.s003.doc]

**Table S3.** Association between SNPs and clinical features in control and newly diagnosed type 2 diabetes subjects.

|  | **Normoglycaemic control** | | | |  | **Newly diagnosed type 2 diabetes** | | | |  |
| --- | --- | --- | --- | --- | --- | --- | --- | --- | --- | --- |
|  |  | | | | **Empirical** |  | | | | **Empirical** |
| **Traits** | **Gene** | **SNP** | ***β*** | ***p*** | ***p*** | **Gene** | **SNP** | ***β*** | ***p*** | ***p*** |
| **Height (cm)** | *MTNR1B* | rs10830963 | -0.00317 | <0.0001 | **0.0030** | *NOTCH2* | rs10923931 | -0.00600 | 0.0347 | 0.6444 |
|  | *TSPAN8/LGR5* | rs7961581 | 0.00196 | 0.0456 | 0.7602 | *JAZF1* | rs864745 | 0.00287 | 0.0217 | 0.4635 |
|  | *GCKR* | rs780094 | 0.00156 | 0.0432 | 0.7283 | *HHEX* | rs1111875 | -0.00253 | 0.0306 | 0.5934 |
| **Weight (kg)** | *ADAMTS9* | rs4607103 | -0.00619 | 0.0374 | 0.6703 | *KCNQ1* | rs2237895 | -0.01586 | 0.0013 | **0.0370** |
|  | *HNF1A* | rs7957197 | -0.07112 | 0.0257 | 0.5205 |  |  |  |  |  |
|  | *GCKR* | rs780094 | 0.00599 | 0.0335 | 0.6204 |  |  |  |  |  |
| **BMI (kg/m^2^)** | *ADAMTS9* | rs4607103 | -0.00572 | 0.0232 | 0.4825 | *KCNQ1* | rs2237895 | -0.01738 | 0.0001 | **0.0070** |
|  | *HNF1A* | rs7957197 | -0.06926 | 0.0102 | 0.2527 | *FTO* | rs8050136 | 0.01336 | 0.0275 | 0.5804 |
|  |  |  |  |  |  | *FTO* | rs9939609 | 0.01294 | 0.0325 | 0.6354 |
| **Waist circumference (cm)** | *JAZF1* | rs864745 | -0.00530 | 0.0399 | 0.7083 | *KCNQ1* | rs2237895 | -0.01055 | 0.0028 | 0.0799 |
|  | *ADAMTS9* | rs4607103 | -0.00623 | 0.0065 | 0.1718 | *FTO* | rs8050136 | 0.01121 | 0.0175 | 0.3926 |
|  | *GCKR* | rs780094 | 0.00434 | 0.0455 | 0.7572 | *FTO* | rs9939609 | 0.01093 | 0.0202 | 0.4316 |
|  |  |  |  |  |  | *HHEX* | rs1111875 | -0.00765 | 0.0276 | 0.5534 |
|  |  |  |  |  |  | *CHCHD9* | rs13292136 | 0.01310 | 0.0130 | 0.3057 |
| **Hip circumference (cm)** | *GCKR* | rs780094 | 0.00323 | 0.0284 | 0.5634 | *GCKR* | rs780094 | -0.00458 | 0.0470 | 0.7363 |
|  |  |  |  |  |  | *KCNQ1* | rs2237895 | -0.00603 | 0.0181 | 0.3886 |
|  |  |  |  |  |  | *FTO* | rs8050136 | 0.00803 | 0.0196 | 0.4166 |
|  |  |  |  |  |  | *FTO* | rs9939609 | 0.00757 | 0.0275 | 0.5385 |
|  |  |  |  |  |  | *ZBED3* | rs4457053 | 0.01082 | 0.0421 | 0.6953 |
|  |  |  |  |  |  | *CDC123/CAMK1D* | rs12779790 | -0.00693 | 0.0188 | 0.4016 |
| **Waist-hip-ratio** | *CDKAL1* | rs7756992 | 0.00370 | 0.0207 | 0.4535 |  |  |  |  |  |
|  | *JAZF1* | rs864745 | -0.00560 | 0.0027 | 0.0709 |  |  |  |  |  |
|  | *ADAMTS9* | rs4607103 | -0.00436 | 0.0084 | 0.1968 |  |  |  |  |  |
| **Systolic blood pressure (mmHg)** | *MTNR1B* | rs10830963 | -0.00487 | 0.0269 | 0.5435 | *MTNR1B* | rs10830963 | 0.00996 | 0.0147 | 0.3337 |
|  | *GCKR* | rs780094 | -0.00646 | 0.0024 | 0.0679 |  |  |  |  |  |
| **Diastolic blood pressure (mmHg)** | *GCKR* | rs780094 | -0.00666 | 0.0025 | 0.0819 | *MTNR1B* | rs10830963 | 0.01328 | 0.0006 | **0.0190** |
|  |  |  |  |  |  | *THADA* | rs7578597 | -0.05617 | 0.0055 | 0.1289 |
| **Fasting plasma glucose (mmol/l)** | *MTNR1B* | rs10830963 | 0.00664 | 0.0046 | 0.1399 | *GCKR* | rs780094 | 0.02462 | 0.0045 | 0.1179 |
|  |  |  |  |  |  | *ZBED3* | rs4457053 | -0.04249 | 0.0326 | 0.6154 |
| **30min plasma glucose (mmol/l)** | *MTNR1B* | rs10830963 | 0.01380 | 0.0046 | 0.1189 | *KCNQ1* | rs2237895 | 0.02540 | 0.0064 | 0.1618 |
|  | *CDKN2BAS* | rs10811661 | 0.01189 | 0.0128 | 0.3237 | *CDC123/CAMK1D* | rs12779790 | 0.02099 | 0.0490 | 0.7403 |
|  | *CDKAL1* | rs7756992 | 0.01669 | 0.0006 | **0.0140** |  |  |  |  |  |
|  | *TCF2* | rs7501939 | 0.01204 | 0.0261 | 0.5385 |  |  |  |  |  |
|  | *CENTD2* | rs1552224 | 0.01841 | 0.0265 | 0.5375 |  |  |  |  |  |
| **2h plasma glucose (mmol/l)** | *CDKAL1* | rs7756992 | 0.01082 | 0.0139 | 0.3307 | *CDKAL1* | rs7756992 | 0.02098 | 0.0311 | 0.6274 |
|  | *TCF7L2* | rs7903146 | 0.02857 | 0.0146 | 0.3417 |  |  |  |  |  |
|  | *CDKN2BAS* | rs10811661 | 0.00933 | 0.0317 | 0.5934 |  |  |  |  |  |
| **Fasting serum insulin (mU/l)** | *CDC123/CAMK1D* | rs12779790 | 0.03182 | 0.0187 | 0.4446 |  |  |  |  |  |
|  | *TP53INP1* | rs896854 | -0.02543 | 0.0193 | 0.4535 |  |  |  |  |  |
|  | *FTO* | rs9939609 | -0.03232 | 0.0413 | 0.7223 |  |  |  |  |  |
| **30min serum insulin (mU/l)** | *CDKAL1* | rs7756992 | -0.04269 | 0.0090 | 0.2547 | *WFS1* | rs10010131 | -0.13260 | 0.0343 | 0.6214 |
|  | *HHEX* | rs1111875 | -0.04054 | 0.0243 | 0.5574 | *JAZF1* | rs864745 | -0.05940 | 0.0439 | 0.7183 |
|  | *CENTD2* | rs1552224 | -0.06977 | 0.0134 | 0.3526 | *CDKN2BAS* | rs10811661 | -0.05332 | 0.0350 | 0.6284 |
|  | *ZFAND6* | rs11634397 | -0.06361 | 0.0201 | 0.4565 |  |  |  |  |  |
| **2h serum insulin (mU/l)** | *THADA* | rs7578597 | -0.20580 | 0.0317 | 0.6004 | *CDKN2BAS* | rs10811661 | -0.05530 | 0.0392 | 0.6843 |
|  | *PPARG* | rs1801282 | 0.08827 | 0.0070 | 0.1648 |  |  |  |  |  |
|  | *ZFAND6* | rs11634397 | -0.05612 | 0.0371 | 0.6404 |  |  |  |  |  |
|  | *GCKR* | rs780094 | 0.03990 | 0.0108 | 0.2687 |  |  |  |  |  |
| **Triglycerides (mmol/l)** | *PPARG* | rs1801282 | 0.03560 | 0.0190 | 0.4086 | *GCKR* | rs780094 | -0.04332 | 0.0082 | 0.2148 |
|  | *NOTCH2* | rs10923931 | -0.04229 | 0.0361 | 0.6563 | *ADAMTS9* | rs4607103 | -0.04230 | 0.0124 | 0.3117 |
|  |  |  |  |  |  | *CHCHD9* | rs13292136 | 0.06043 | 0.0270 | 0.5405 |
| **Total cholesterol (mmol/l)** | *CDKAL1* | rs7756992 | -0.01124 | 0.0075 | 0.1958 | *CDKN2BAS* | rs10811661 | 0.01342 | 0.0257 | 0.5055 |
|  | *ZBED3* | rs4457053 | 0.02585 | 0.0072 | 0.1898 |  |  |  |  |  |
| **High density lipoprotein** | *MTNR1B* | rs10830963 | 0.01213 | 0.0233 | 0.5065 | *MTNR1B* | rs10830963 | 0.02018 | 0.0079 | 0.2068 |
| **-cholesterol (mmol/l)** | *ZBED3* | rs4457053 | 0.02612 | 0.0308 | 0.6164 |  |  |  |  |  |
|  | *HNF1A* | rs7957197 | 0.11580 | 0.0464 | 0.7602 |  |  |  |  |  |
|  | *GCKR* | rs780094 | 0.01233 | 0.0172 | 0.4046 |  |  |  |  |  |
| **Low density lipoprotein** | *CDKAL1* | rs7756992 | -0.02094 | 0.0054 | 0.1628 | *CDKN2BAS* | rs10811661 | 0.02456 | 0.0276 | 0.5455 |
| **-cholesterol (mmol/l)** | *ZBED3* | rs4457053 | 0.03689 | 0.0317 | 0.6124 | *TCF2* | rs7501939 | 0.02566 | 0.0323 | 0.6064 |
| **HOMA-IR** | *CDC123/CAMK1D* | rs12779790 | 0.03139 | 0.0252 | 0.5335 | *TSPAN8/LGR5* | rs7961581 | -0.07315 | 0.0113 | 0.2687 |
|  | *TP53INP1* | rs896854 | -0.02440 | 0.0303 | 0.5804 | *BCL11A* | rs243021 | -0.04955 | 0.0455 | 0.7303 |
|  | *FTO* | rs9939609 | -0.03307 | 0.0439 | 0.7333 | *ZBED3* | rs4457053 | 0.11750 | 0.0325 | 0.5944 |
| **HOMA-B (%)** | *ZFAND6* | rs11634397 | 0.04654 | 0.0497 | 0.7662 |  |  |  |  |  |
| **ISIm** | *CDC123/CAMK1D* | rs12779790 | -0.03909 | 0.0056 | 0.1369 |  |  |  |  |  |
|  | *HHEX* | rs1111875 | 0.02944 | 0.0124 | 0.2927 |  |  |  |  |  |
|  | *TP53INP1* | rs896854 | 0.02385 | 0.0365 | 0.6474 |  |  |  |  |  |
|  | *GCKR* | rs780094 | -0.02561 | 0.0144 | 0.3407 |  |  |  |  |  |
| **Insulinogenic index (∆I_30_/∆G_30_)** | *CDKN2BAS* | rs10811661 | -0.05004 | 0.0401 | 0.7033 | *CDKN2BAS* | rs10811661 | -0.09431 | 0.0373 | 0.6533 |
|  | *CDKAL1* | rs7756992 | -0.06429 | 0.0094 | 0.2478 | *KCNQ1* | rs2237895 | -0.14890 | 0.0030 | 0.0979 |
|  | *HHEX* | rs1111875 | -0.08313 | 0.0022 | 0.0709 |  |  |  |  |  |
|  | *ADAMTS9* | rs4607103 | -0.07218 | 0.0048 | 0.1489 |  |  |  |  |  |
|  | *ZFAND6* | rs11634397 | -0.10952 | 0.0083 | 0.2178 |  |  |  |  |  |
| **∆I_30_/∆G_30_/HOMA-IR** | *CDKN2BAS* | rs10811661 | -0.05659 | 0.0220 | 0.4935 | *CDKN2BAS* | rs10811661 | -0.10890 | 0.0251 | 0.5195 |
|  | *CDKAL1* | rs7756992 | -0.06454 | 0.0101 | 0.2547 | *KCNQ1* | rs2237895 | -0.14050 | 0.0088 | 0.2238 |
|  | *HHEX* | rs1111875 | -0.06937 | 0.0117 | 0.2977 |  |  |  |  |  |
|  | *ADAMTS9* | rs4607103 | -0.07709 | 0.0029 | 0.0899 |  |  |  |  |  |
|  | *ZFAND6* | rs11634397 | -0.10813 | 0.0101 | 0.2547 |  |  |  |  |  |

*β* value is reported for the allele with higher type 2 diabetes risk as previously reported in Caucasians using an additive model in multi-variate linear regression adjusted for region, gender and age, with BMI when appropriate.

Associations with *p* value < 0.05 are shown in the table. Empirical *p* values were calculated through 1,000 permutations. Empirical *p* values < 0.05 are shown in bold.
