# Supplementary material for: Association of Genetic Variants with Isolated Fasting Hyperglycaemia and Isolated Postprandial Hyperglycaemia in a Han Chinese Population
Source: PLoS One. 2013 Aug 19;8(8):e71399. doi: 10.1371/journal.pone.0071399 (PMC3747192; doi:10.1371/journal.pone.0071399)
Supplement: Table S4 — SNPs did not show significant association with isolated fasting hyperglycemia in Hans. a Risk alleles for type 2 diabetes in the Caucasian descent population are denoted in bold. OR and 95% CI are reported for the allele with higher type 2 diabetes risk previously reported for Caucasians using χ2 or an additive model in logistic regression. b Comparison of the allelic distribution between isolated fasting hyperglycemia and controls. c Comparison of the genotype distribution between isolated fasting hyperglycemia and controls after adjusting for region, age and gender. d Comparison of the genotype distribution between isolated fasting hyperglycemia and controls after adjusting for region, age, gender and BMI. We failed to compare genotype distribution between isolated fasting hyperglycemia and controls at rs7957197 because its minor allele frequency of present samples was very low. Empirical p values were calculated through 1,000 permutations. p values<0.05 are shown in bold. (DOC) [file pone.0071399.s004.doc]

**Table S4** SNPs did not show significant association with isolated fasting hyperglycemia in Hans.

|  |  | **Minor/major** | | **Allelic** | **Genotypic** | **Genotypic** |
| --- | --- | --- | --- | --- | --- | --- |
| **Gene** | **SNP** | **allele^a^** | | **association^b^** | **association^c^** | **association^d^** |
| *CDKAL1* | rs7756992 | A/**G** | OR (95%CI) | 0.958 (0.820,1.120) | 0.961 (0.821,1.126) | 0.989 (0.837,1.169) |
|  |  |  | *p* | 0.5894 | 0.6239 | 0.9002 |
|  |  |  | Empirical *p* | 1.0000 |  |  |
| *TP53INP1* | rs896854 | **A**/G | OR (95%CI) | 1.115 (0.948,1.311) | 1.114 (0.944,1.316) | 1.068 (0.895,1.276) |
|  |  |  | *p* | 0.1877 | 0.2025 | 0.4645 |
|  |  |  | Empirical *p* | 0.9970 |  |  |
| *PRC1* | rs8042680 | C/**A** | OR (95%CI) | 1.514 (0.740,3.095) | 1.559 (0.760,3.198) | 1.735 (0.792,3.802) |
|  |  |  | *p* | 0.2529 | 0.2254 | 0.1686 |
|  |  |  | Empirical *p* | 1.0000 |  |  |
| *HHEX* | rs1111875 | **G**/A | OR (95%CI) | 1.086 (0.915,1.288) | 1.077 (0.907,1.278) | 1.076 (0.895,1.293) |
|  |  |  | *p* | 0.3441 | 0.4000 | 0.4356 |
|  |  |  | Empirical *p* | 1.0000 |  |  |
| *TCF2* | rs7501939 | **T**/C | OR (95%CI) | 1.087 (0.914,1.293) | 1.079 (0.907,1.283) | 1.086 (0.904,1.306) |
|  |  |  | *p* | 0.3469 | 0.3908 | 0.3765 |
|  |  |  | Empirical *p* | 1.0000 |  |  |
| *WFS1* | rs10010131 | A/**G** | OR (95%CI) | 1.123 (0.759,1.662) | 1.144 (0.770,1.700) | 1.138 (0.753,1.720) |
|  |  |  | *p* | 0.5607 | 0.5055 | 0.5390 |
|  |  |  | Empirical *p* | 1.0000 |  |  |
| *CDC123/CAMK1D* | rs12779790 | **G**/A | OR (95%CI) | 1.015 (0.823,1.253) | 1.018 (0.825,1.256) | 1.041 (0.833,1.302) |
|  |  |  | *p* | 0.8866 | 0.8654 | 0.7241 |
|  |  |  | Empirical *p* | 1.0000 |  |  |
| *MTNRIB* | rs10830963 | **G**/C | OR (95%CI) | 1.087 (0.928,1.273) | 1.088 (0.926,1.278) | 1.057 (0.891,1.254) |
|  |  |  | *p* | 0.3000 | 0.3043 | 0.5255 |
|  |  |  | Empirical *p* | 1.0000 |  |  |
| *TSPAN8/LGR5* | rs7961581 | **C**/T | OR (95%CI) | 1.085 (0.897,1.313) | 1.086 (0.896,1.317) | 1.075 (0.878,1.318) |
|  |  |  | *p* | 0.4023 | 0.4020 | 0.4838 |
|  |  |  | Empirical *p* | 1.0000 |  |  |
| *THADA* | rs7578597 | C/**T** | OR (95%CI) | 0.662 (0.301,1.456) | 0.617 (0.278,1.371) | 0.633 (0.269,1.489) |
|  |  |  | *p* | 0.3015 | 0.2360 | 0.2945 |
|  |  |  | Empirical *p* | 1.0000 |  |  |
| *JAZF1* | rs864745 | G/**A** | OR (95%CI) | 0.886 (0.741,1.060) | 0.902 (0.753,1.080) | 0.951 (0.786,1.149) |
|  |  |  | *p* | 0.1855 | 0.2623 | 0.6024 |
|  |  |  | Empirical *p* | 0.9970 |  |  |
| *PPARG* | rs1801282 | G/**C** | OR (95%CI) | 1.042 (0.754,1.441) | 1.039 (0.749,1.441) | 0.963 (0.684,1.354) |
|  |  |  | *p* | 0.8046 | 0.8173 | 0.8272 |
|  |  |  | Empirical *p* | 1.0000 |  |  |
| *ADAMTS9* | rs4607103 | T/**C** | OR (95%CI) | 0.997 (0.849,1.172) | 0.992 (0.842,1.167) | 1.040 (0.875,1.235) |
|  |  |  | *p* | 0.9726 | 0.9184 | 0.6563 |
|  |  |  | Empirical *p* | 1.0000 |  |  |
| *NOTCH2* | rs10923931 | **T**/G | OR (95%CI) | 0.892 (0.569,1.399) | 0.950 (0.606,1.492) | 0.916 (0.571,1.469) |
|  |  |  | *p* | 0.6181 | 0.8249 | 0.7162 |
|  |  |  | Empirical *p* | 1.0000 |  |  |
| *BCL11A* | rs243021 | C/**T** | OR (95%CI) | 0.966 (0.818,1.142) | 0.960 (0.812,1.135) | 0.953 (0.798,1.138) |
|  |  |  | *p* | 0.6875 | 0.6329 | 0.5948 |
|  |  |  | Empirical *p* | 1.0000 |  |  |
| *ZBED3* | rs4457053 | **G**/A | OR (95%CI) | 1.062 (0.748,1.506) | 1.055 (0.742,1.502) | 1.068 (0.734,1.554) |
|  |  |  | *p* | 0.7371 | 0.7641 | 0.7306 |
|  |  |  | Empirical *p* | 1.0000 |  |  |
| *KLF14* | rs972283 | A/**G** | OR (95%CI) | 0.967 (0.810,1.155) | 0.976 (0.820,1.163) | 0.999 (0.831,1.201) |
|  |  |  | *p* | 0.7123 | 0.7898 | 0.9923 |
|  |  |  | Empirical *p* | 1.0000 |  |  |
| *CHCHD9* | rs13292136 | T/**C** | OR (95%CI) | 0.826 (0.620,1.102) | 0.821 (0.614,1.098) | 0.797 (0.585,1.085) |
|  |  |  | *p* | 0.1928 | 0.1843 | 0.1497 |
|  |  |  | Empirical *p* | 0.9980 |  |  |
| *CENTD2* | rs1552224 | G/**T** | OR (95%CI) | 1.157 (0.867,1.542) | 1.144 (0.858,1.526) | 1.084 (0.796,1.477) |
|  |  |  | *p* | 0.3211 | 0.3602 | 0.6079 |
|  |  |  | Empirical *p* | 1.0000 |  |  |
| *HNF1A* | rs7957197 | A/**T** | OR (95%CI) | / | / | / |
|  |  |  | *p* | / | / | / |
|  |  |  | Empirical *p* | / |  |  |
| *ZFAND6* | rs11634397 | **G**/A | OR (95%CI) | 0.810 (0.608,1.079) | 0.788 (0.589,1.053) | 0.789 (0.579,1.075) |
|  |  |  | *p* | 0.1497 | 0.1068 | 0.1336 |
|  |  |  | Empirical *p* | 0.9900 |  |  |

^a^ Risk alleles for type 2 diabetes in the Caucasian descent population are denoted in bold. OR and 95% CI are reported for the allele with higher type 2 diabetes risk previously reported for Caucasians using χ2 or an additive model in logistic regression.

^b^ Comparison of the allelic distribution between isolated fasting hyperglycemia and controls.

^c^ Comparison of the genotype distribution between isolated fasting hyperglycemia and controls after adjusting for region, age and gender.

^d^ Comparison of the genotype distribution between isolated fasting hyperglycemia and controls after adjusting for region, age, gender and BMI.

We failed to compare genotype distribution between isolated fasting hyperglycemia and controls at rs7957197 because its minor allele frequency of present samples was very low.

Empirical *p* values were calculated through 1,000 permutations. *p* values < 0.05 are shown in bold.
