# Supplementary material for: Association of Genetic Variants with Isolated Fasting Hyperglycaemia and Isolated Postprandial Hyperglycaemia in a Han Chinese Population
Source: PLoS One. 2013 Aug 19;8(8):e71399. doi: 10.1371/journal.pone.0071399 (PMC3747192; doi:10.1371/journal.pone.0071399)
Supplement: Table S5 — SNPs did not show significant association with isolated postprandial hyperglycemia in Hans. a Risk alleles for type 2 diabetes in the Caucasian descent population are denoted in bold. OR and 95% CI are reported for the allele with higher type 2 diabetes risk previously reported for Caucasians using χ2 or an additive model in logistic regression. b Comparison of the allelic distribution between isolated postprandial hyperglycemia and controls. c Comparison of the genotype distribution between isolated postprandial hyperglycemia and controls after adjusting for region, age and gender. d Comparison of the genotype distribution between isolated postprandial hyperglycemia and controls after adjusting for region, age, gender and BMI. Empirical p values were calculated through 1,000 permutations. p values <0.05 are shown in bold. (DOC) [file pone.0071399.s005.doc]

**Table S5** SNPs did not show significant association with isolated postprandial hyperglycemia in Hans.

|  |  | **Minor/major** | | **Allelic** | **Genotypic** | **Genotypic** |
| --- | --- | --- | --- | --- | --- | --- |
| **Gene** | **SNP** | **allele^a^** | | **association^b^** | **association^c^** | **association^d^** |
| *CDKN2BAS* | rs10811661 | C/**T** | OR (95%CI) | 1.067 (0.962,1.184) | 1.083 (0.973,1.205) | 1.079 (0.959,1.214) |
|  |  |  | *p* | 0.2207 | 0.1448 | 0.2072 |
|  |  |  | Empirical *p* | 1.0000 |  |  |
| *FTO* | rs8050136 | **A**/C | OR (95%CI) | 0.947 (0.802,1.118) | 0.926 (0.782,1.098) | 0.876 (0.728,1.054) |
|  |  |  | *p* | 0.5202 | 0.3777 | 0.1600 |
|  |  |  | Empirical *p* | 1.0000 |  |  |
| *FTO* | rs9939609 | **A**/T | OR (95%CI) | 0.968 (0.822,1.141) | 0.954 (0.806,1.129) | 0.902 (0.750,1.083) |
|  |  |  | *p* | 0.7007 | 0.5846 | 0.2688 |
|  |  |  | Empirical *p* | 1.0000 |  |  |
| *TCF2* | rs7501939 | **T**/C | OR (95%CI) | 1.102 (0.983,1.237) | 1.124 (0.999,1.265) | 1.114 (0.978,1.270) |
|  |  |  | *p* | 0.0967 | 0.0523 | 0.1045 |
|  |  |  | Empirical *p* | 0.9461 |  |  |
| *WFS1* | rs10010131 | A/**G** | OR (95%CI) | 1.045 (0.813,1.344) | 1.084 (0.835,1.406) | 1.198 (0.899,1.594) |
|  |  |  | *p* | 0.7298 | 0.5434 | 0.2171 |
|  |  |  | Empirical *p* | 1.0000 |  |  |
| *CDC123/CAMK1D* | rs12779790 | **G**/A | OR (95%CI) | 1.006 (0.875,1.157) | 1.005 (0.871,1.159) | 1.041 (0.890,1.219) |
|  |  |  | *p* | 0.9305 | 0.9490 | 0.6121 |
|  |  |  | Empirical *p* | 1.0000 |  |  |
| *MTNRIB* | rs10830963 | **G**/C | OR (95%CI) | 1.060 (0.955,1.177) | 1.045 (0.938,1.164) | 1.065 (0.946,1.198) |
|  |  |  | *p* | 0.2757 | 0.4261 | 0.2998 |
|  |  |  | Empirical *p* | 1.0000 |  |  |
| *TSPAN8/LGR5* | rs7961581 | **C**/T | OR (95%CI) | 0.923 (0.809,1.053) | 0.929 (0.811,1.065) | 0.892 (0.768,1.035) |
|  |  |  | *p* | 0.2340 | 0.2897 | 0.1306 |
|  |  |  | Empirical *p* | 1.0000 |  |  |
| *THADA* | rs7578597 | C/**T** | OR (95%CI) | 0.688 (0.400,1.131) | 0.657 (0.374,1.155) | 0.661 (0.352,1.106) |
|  |  |  | *p* | 0.1748 | 0.1445 | 0.1991 |
|  |  |  | Empirical *p* | 0.9980 |  |  |
| *JAZF1* | rs864745 | G/**A** | OR (95%CI) | 0.920 (0.816,1.037) | 0.918 (0.812,1.039) | 0.984 (0.859,1.127) |
|  |  |  | *p* | 0.1723 | 0.1763 | 0.8159 |
|  |  |  | Empirical *p* | 0.9980 |  |  |
| *PPARG* | rs1801282 | G/**C** | OR (95%CI) | 0.994 (0.805,1.228) | 0.968 (0.779,1.204) | 1.049 (0.821,1.339) |
|  |  |  | *p* | 0.9561 | 0.7722 | 0.7042 |
|  |  |  | Empirical *p* | 1.0000 |  |  |
| *ADAMTS9* | rs4607103 | T/**C** | OR (95%CI) | 1.048 (0.942,1.167) | 1.051 (0.941,1.175) | 1.098 (0.972,1.241) |
|  |  |  | *p* | 0.3886 | 0.3794 | 0.1341 |
|  |  |  | Empirical *p* | 1.0000 |  |  |
| *NOTCH2* | rs10923931 | **T**/G | OR (95%CI) | 1.113 (0.848,1.461) | 1.129 (0.857,1.489) | 1.084 (0.800,1.471) |
|  |  |  | *p* | 0.4412 | 0.3879 | 0.6025 |
|  |  |  | Empirical *p* | 1.0000 |  |  |
| *BCL11A* | rs243021 | C/**T** | OR (95%CI) | 0.977 (0.875,1.091) | 0.977 (0.871,1.095) | 0.946 (0.835,1.073) |
|  |  |  | *p* | 0.6802 | 0.6836 | 0.3872 |
|  |  |  | Empirical *p* | 1.0000 |  |  |
| *ZBED3* | rs4457053 | **G**/A | OR (95%CI) | 1.175 (0.939,1.470) | 1.149 (0.910,1.451) | 1.173 (0.905,1.519) |
|  |  |  | *p* | 0.1596 | 0.2436 | 0.2285 |
|  |  |  | Empirical *p* | 0.9960 |  |  |
| *KLF14* | rs972283 | A/**G** | OR (95%CI) | 1.039 (0.926,1.166) | 1.035 (0.920,1.163) | 1.065 (0.937,1.211) |
|  |  |  | *p* | 0.5110 | 0.5680 | 0.3369 |
|  |  |  | Empirical *p* | 1.0000 |  |  |
| *CHCHD9* | rs13292136 | T/**C** | OR (95%CI) | 1.062 (0.893,1.263) | 1.052 (0.879,1.259) | 1.018 (0.835,1.241) |
|  |  |  | *p* | 0.4984 | 0.5809 | 0.8603 |
|  |  |  | Empirical *p* | 1.0000 |  |  |
| *CENTD2* | rs1552224 | G/**T** | OR (95%CI) | 1.128 (0.935,1.361) | 1.123 (0.923,1.365) | 1.079 (0.870,1.337) |
|  |  |  | *p* | 0.2087 | 0.2460 | 0.4901 |
|  |  |  | Empirical *p* | 1.0000 |  |  |
| *HNF1A* | rs7957197 | A/**T** | OR (95%CI) | 0.864 (0.253,2.951) | 0.829 (0.238,2.889) | 0.984 (0.227,4.276) |
|  |  |  | *p* | 0.8151 | 0.7684 | 0.9831 |
|  |  |  | Empirical *p* | 1.0000 |  |  |
| *ZFAND6* | rs11634397 | **G**/A | OR (95%CI) | 1.056 (0.889,1.255) | 1.046 (0.875,1.251) | 1.039 (0.852,1.268) |
|  |  |  | *p* | 0.5350 | 0.6186 | 0.7066 |
|  |  |  | Empirical *p* | 1.0000 |  |  |

^a^ Risk alleles for type 2 diabetes in the Caucasian descent population are denoted in bold. OR and 95% CI are reported for the allele with higher type 2 diabetes risk previously reported for Caucasians using χ2 or an additive model in logistic regression.

^b^ Comparison of the allelic distribution between isolated postprandial hyperglycemia and controls.

^c^ Comparison of the genotype distribution between isolated postprandial hyperglycemia and controls after adjusting for region, age and gender.

^d^ Comparison of the genotype distribution between isolated postprandial hyperglycemia and controls after adjusting for region, age, gender and BMI.

Empirical *p* values were calculated through 1,000 permutations. *p* values < 0.05 are shown in bold.
