# Supplementary material for: Association of Genetic Variants with Isolated Fasting Hyperglycaemia and Isolated Postprandial Hyperglycaemia in a Han Chinese Population
Source: PLoS One. 2013 Aug 19;8(8):e71399. doi: 10.1371/journal.pone.0071399 (PMC3747192; doi:10.1371/journal.pone.0071399)
Supplement: Table S6 — SNPs did not show significant differences in risk allele frequency or genotype distribution between isolated fasting hyperglycemia and isolated postprandial hyperglycemia. a Risk alleles for type 2 diabetes in Caucasians are denoted in bold. Allelic frequencies between isolated fasting hyperglycemia and isolated postprandial hyperglycemia were compared using χ2 test. Genotype distributions are shown as the counts of three genotypes (BB, Bb, bb). B, risk allele; b, non-risk allele. Genotype distributions between isolated fasting hyperglycemia and isolated postprandial hyperglycemia were compared using Cochran-Armitage trend test. p values <0.05 are shown in bold. IFH, isolated fasting hyperglycemia; IPH, isolated postprandial hyperglycemia. (DOC) [file pone.0071399.s006.doc]

**Table S6** SNPs did not show significant differences in risk allele frequency or genotype distribution between isolated fasting hyperglycemia and isolated postprandial hyperglycemia.

|  |  | | **Risk allele** | | **χ^2^ test of risk** | | **Genotype distribution** | | **Cochran-Armitage trend test** | |
| --- | --- | --- | --- | --- | --- | --- | --- | --- | --- | --- |
|  | **Minor/major** | | **frequency** | | **allele frequency** | | **(BB/Bb/bb)** | | **of genotype distribution** | |
| **Gene** | **SNP** | **allele^a^** | **IFH** | **IPH** | **χ^2^** | ***p*** | **IFH** | **IPH** | **Z** | **Two-tails *p*** |
| *TCF7L2* | rs7903146 | **T**/C | 0.054 | 0.054 | 0.0032 | 0.9550 | 1/35/305 | 2/88/767 | -0.0566 | 0.9548 |
| *KCNQ1* | rs2237895 | **C**/A | 0.357 | 0.348 | 0.1542 | 0.6946 | 32/146/116 | 100/343/337 | -0.3926 | 0.6946 |
| *CDKN2BAS* | rs10811661 | C/**T** | 0.569 | 0.542 | 1.4124 | 0.2347 | 118/151/71 | 241/453/168 | -1.1975 | 0.2311 |
| *TP53INP1* | rs896854 | **A**/G | 0.368 | 0.367 | 0.0035 | 0.9528 | 40/171/130 | 118/397/348 | -0.0597 | 0.9524 |
| *PRC1* | rs8042680 | C/**A** | 0.988 | 0.990 | 0.0806 | 0.7765 | 333/8/0 | 847/18/0 | 0.2855 | 0.7753 |
| *HHEX* | rs1111875 | **G**/A | 0.299 | 0.302 | 0.0315 | 0.8592 | 29/145/166 | 80/358/419 | 0.1772 | 0.8593 |
| *TCF2* | rs7501939 | **T**/C | 0.282 | 0.284 | 0.0198 | 0.8881 | 34/124/183 | 78/336/451 | 0.1366 | 0.8913 |
| *WFS1* | rs10010131 | A/**G** | 0.959 | 0.956 | 0.1014 | 0.7502 | 313/28/0 | 791/70/3 | -0.3160 | 0.7520 |
| *CDC123/CAMK1D* | rs12779790 | **G**/A | 0.166 | 0.165 | 0.0055 | 0.9407 | 11/91/238 | 29/226/606 | -0.0729 | 0.9419 |
| *MTNRIB* | rs10830963 | **G**/C | 0.431 | 0.425 | 0.0758 | 0.7831 | 49/194/96 | 166/399/296 | -0.2767 | 0.7820 |
| *TSPAN8/LGR5* | rs7961581 | **C**/T | 0.215 | 0.189 | 2.0860 | 0.1487 | 11/124/205 | 28/269/564 | -1.4725 | 0.1409 |
| *THADA* | rs7578597 | C/**T** | 0.990 | 0.990 | 0.0074 | 0.9316 | 333/7/0 | 841/17/0 | 0.0863 | 0.9312 |
| *JAZF1* | rs864745 | G/**A** | 0.741 | 0.748 | 0.1301 | 0.7183 | 185/131/22 | 478/328/52 | 0.3631 | 0.7165 |
| *PPARG* | rs1801282 | G/**C** | 0.938 | 0.935 | 0.0627 | 0.8023 | 299/40/1 | 753/103/4 | 1.7528 | 0.0796 |
| *ADAMTS9* | rs4607103 | T/**C** | 0.625 | 0.636 | 0.2868 | 0.5922 | 133/160/48 | 351/395/116 | 0.5337 | 0.5935 |
| *NOTCH2* | rs10923931 | **T**/G | 0.031 | 0.038 | 0.7574 | 0.3841 | 0/21/319 | 3/60/800 | 0.8559 | 0.3921 |
| *BCL11A* | rs243021 | C/**T** | 0.675 | 0.677 | 0.0128 | 0.9100 | 157/146/38 | 401/369/95 | 0.1117 | 0.9111 |
| *ZBED3* | rs4457053 | **G**/A | 0.053 | 0.058 | 0.2555 | 0.6132 | 1/34/304 | 4/92/760 | 0.5011 | 0.6163 |
| *KLF14* | rs972283 | A/**G** | 0.730 | 0.715 | 0.4958 | 0.4814 | 183/123/29 | 446/340/75 | -0.6901 | 0.4901 |
| *CHCHD9* | rs13292136 | T/**C** | 0.921 | 0.900 | 2.3725 | 0.1235 | 287/52/1 | 699/154/9 | -1.5450 | 0.1223 |
| *CENTD2* | rs1552224 | G/**T** | 0.921 | 0.919 | 0.0224 | 0.8811 | 290/48/3 | 727/134/3 | -0.1511 | 0.8799 |
| *HNF1A* | rs7957197 | A/**T** | 1.000 | 0.998 | 1.1841 | 0.2765 | 341/0/0 | 862/3/0 | -1.0889 | 0.2762 |
| *ZFAND6* | rs11634397 | **G**/A | 0.079 | 0.101 | 2.6687 | 0.1023 | 2/50/289 | 12/150/701 | 1.6088 | 0.1077 |

^a^ Risk alleles for type 2 diabetes in Caucasians are denoted in bold.

Allelic frequencies between isolated fasting hyperglycemia and isolated postprandial hyperglycemia were compared using χ2 test.

Genotype distributions are shown as the counts of three genotypes (BB, Bb, bb). B, risk allele; b, non-risk allele. Genotype distributions between isolated fasting hyperglycemia and isolated postprandial hyperglycemia were compared using Cochran-Armitage trend test.

*p* values < 0.05 are shown in bold.

IFH, isolated fasting hyperglycemia; IPH, isolated postprandial hyperglycemia.
