# Supplementary material for: Association of Genetic Variants with Isolated Fasting Hyperglycaemia and Isolated Postprandial Hyperglycaemia in a Han Chinese Population
Source: PLoS One. 2013 Aug 19;8(8):e71399. doi: 10.1371/journal.pone.0071399 (PMC3747192; doi:10.1371/journal.pone.0071399)
Supplement: Table S7 — Power of the association studies. a Risk alleles for type 2 diabetes in Caucasians are denoted in bold. b ORs and 95% CIs reported by reference studies (Ref.). c Power to detect the association between SNPs and newly diagnosed type 2 diabetes was estimated under the additive model and given the reported OR(95%CI), sample size and α = 0.05 (two-sided). d Power to detect the association between SNPs and isolated fasting hyperglycaemia was estimated under the additive model and given the reported OR(95%CI), sample size and α = 0.05 (two-sided). e Power to detect the association between SNPs and isolated postprandial hyperglycaemia was estimated under the additive model and given the reported OR(95%CI), sample size and α = 0.05 (two-sided). IFH, isolated fasting hyperglycemia; IPH, isolated postprandial hyperglycemia. (DOC) [file pone.0071399.s007.doc]

**Table S7**. Power of the association studies.

|  |  |  |  |  |  |  | **Newly diagnosed** | |  |  |  |  |
| --- | --- | --- | --- | --- | --- | --- | --- | --- | --- | --- | --- | --- |
|  | **Minor/major** | | **Reported** |  | **Risk allele** |  | **type 2 diabetes** | |  |  |  |  |
| **Gene** | **SNP** | **allele^a^** | **OR (95%CI)^b^** | **Ref.** | **frequency** | **Control (*n*)** | **(*n*)** | **IFH (*n*)** | **IPH (*n*)** | **Power^c^** | **Power^d^** | **Power^e^** |
| *TCF7L2* | rs7903146 | **T**/C | 1.37 (1.31,1.43) | 15 | 0.036 | 4232 | 2392 | 341 | 857 | 93.21% | 36.46% | 67.03% |
| *KCNQ1* | rs2237895 | **C**/A | 1.32 (1.20,1.45) | 17 | 0.316 | 4107 | 2137 | 294 | 780 | 99.99% | 87.46% | 99.77% |
| *CDKN2BAS* | rs10811661 | C/**T** | 1.20 (1.14,1.25) | 15 | 0.526 | 4238 | 2402 | 340 | 862 | 99.89% | 62.27% | 92.91% |
| *FTO* | rs8050136 | **A**/C | 1.17 (1.12,1.22) | 12 | 0.114 | 4247 | 2405 | 340 | 862 | 81.02% | 25.51% | 49.74% |
| *FTO* | rs9939609 | **A**/T | 1.27 (1.16,1.37) | 20 | 0.114 | 4242 | 2405 | 341 | 864 | 99.22% | 52.36% | 85.69% |
| *GCKR* | rs780094 | **G**/A | 1.16 (1.08,1.24) | 33 | 0.475 | 4245 | 2404 | 340 | 864 | 98.41% | 46.08% | 80.24% |
| *CDKAL1* | rs7756992 | A/**G** | 1.25 (1.11,1.40) | 13 | 0.521 | 4239 | 2399 | 341 | 860 | 99.99% | 79.49% | 98.71% |
| *TP53INP1* | rs896854 | **A**/G | 1.06 (1.04,1.09) | 18 | 0.343 | 4245 | 2403 | 341 | 863 | 33.69% | 10.76% | 18.35% |
| *PRC1* | rs8042680 | C/**A** | 1.07 (1.05,1.09) | 18 | 0.982 | 4250 | 2408 | 341 | 865 | 7.81% | 5.56% | 6.29% |
| *HHEX* | rs1111875 | **G**/A | 1.13 (1.08,1.17) | 15 | 0.282 | 4232 | 2393 | 340 | 857 | 86.75% | 28.88% | 55.81% |
| *TCF2* | rs7501939 | **T**/C | 1.10 (1.06,1.15) | 9 | 0.265 | 4240 | 2408 | 341 | 865 | 65.31% | 18.83% | 36.48% |
| *WFS1* | rs10010131 | A/**G** | 1.11 (1.08,1.16) | 8 | 0.954 | 4250 | 2407 | 341 | 864 | 22.03% | 8.32% | 12.72% |
| *CDC123/CAMK1D* | rs12779790 | **G**/A | 1.11 (1.07,1.14) | 16 | 0.164 | 4236 | 2399 | 340 | 861 | 58.38% | 16.78% | 31.96% |
| *MTNRIB* | rs10830963 | **G**/C | 1.09 (1.05,1.12) | 14 | 0.41 | 4226 | 2401 | 339 | 861 | 65.27% | 18.71% | 36.31% |
| *TSPAN8/LGR5* | rs7961581 | **C**/T | 1.09 (1.06,1.12) | 16 | 0.201 | 4240 | 2401 | 340 | 861 | 48.96% | 14.24% | 26.33% |
| *THADA* | rs7578597 | C/**T** | 1.15 (1.10,1.20) | 16 | 0.993 | 4241 | 2397 | 340 | 858 | 9.58% | 5.90% | 7.08% |
| *JAZF1* | rs864745 | G/**A** | 1.10 (1.07,1.13) | 16 | 0.764 | 4227 | 2392 | 338 | 858 | 59.90% | 16.92% | 32.53% |
| *PPARG* | rs1801282 | G/**C** | 1.14 (1.08,1.20) | 15 | 0.936 | 4246 | 2400 | 340 | 860 | 40.92% | 12.14% | 21.68% |
| *ADAMTS9* | rs4607103 | T/**C** | 1.09 (1.06,1.12) | 16 | 0.625 | 4234 | 2403 | 341 | 862 | 63.23% | 18.03% | 34.80% |
| *NOTCH2* | rs10923931 | **T**/G | 1.13 (1.08,1.17) | 16 | 0.034 | 4247 | 2401 | 340 | 863 | 24.20% | 8.88% | 13.94% |
| *BCL11A* | rs243021 | C/**T** | 1.08 (1.06,1.10) | 18 | 0.682 | 4243 | 2407 | 341 | 865 | 50.53% | 14.50% | 27.06% |
| *ZBED3* | rs4457053 | **G**/A | 1.08 (1.06,1.11) | 18 | 0.05 | 4216 | 2395 | 339 | 856 | 15.61% | 7.14% | 9.89% |
| *KLF14* | rs972283 | A/**G** | 1.07 (1.05,1.10) | 18 | 0.723 | 4231 | 2391 | 335 | 861 | 38.30% | 11.60% | 20.58% |
| *CHCHD9* | rs13292136 | T/**C** | 1.11 (1.07,1.15) | 18 | 0.905 | 4248 | 2404 | 340 | 862 | 38.34% | 11.61% | 20.45% |
| *CENTD2* | rs1552224 | G/**T** | 1.14 (1.11,1.17) | 18 | 0.91 | 4245 | 2408 | 341 | 864 | 52.71% | 14.91% | 28.07% |
| *HNF1A* | rs7957197 | A/**T** | 1.07 (1.05,1.10) | 18 | 0.998 | 4235 | 2408 | 341 | 865 | 5.31% | 5.06% | 5.14% |
| *ZFAND6* | rs11634397 | **G**/A | 1.06 (1.04,1.08) | 18 | 0.096 | 4241 | 2404 | 341 | 863 | 16.06% | 7.22% | 10.10% |

^a^ Risk alleles for type 2 diabetes in Caucasians are denoted in bold.

^b^ ORs and 95% CIs reported by reference studies (Ref.).

^c^ Power to detect the association between SNPs and newly diagnosed type 2 diabetes was estimated under the additive model and given the reported OR(95%CI), sample size and α=0.05 (two-sided).

^d^ Power to detect the association between SNPs and isolated fasting hyperglycaemia was estimated under the additive model and given the reported OR(95%CI), sample size and α=0.05 (two-sided).

^e^ Power to detect the association between SNPs and isolated postprandial hyperglycaemia was estimated under the additive model and given the reported OR(95%CI), sample size and α=0.05 (two-sided).

IFH, isolated fasting hyperglycemia; IPH, isolated postprandial hyperglycemia.
